# Influence of Mechanochemical Pretreatment on the Bioavailability of Mannan from Macauba Seed Cake

**DOI:** 10.1021/acsomega.5c12892

**Published:** 2026-06-13

**Authors:** Michelle R. C. Fortunato, Rosane A. S. San Gil, Leandro B. Borre, Ricardo S. S. Teixeira

**Affiliations:** † Institute of Chemistry, Solid State NMR Laboratory, 28125 Universidade Federal do Rio de Janeiro, Av. Athos da Silveira Ramos, n° 149, Bloco A—6° andar—Lab. 605—Centro de Tecnologia—Cidade Universitária—Ilha do Fundão, Rio de Janeiro CEP 21941-909, Brazil; ‡ Institute of Chemistry, Bioethanol Laboratory, Universidade Federal do Rio de Janeiro, Av. Pedro Calmon, S/N—Bloco P, P4, Cidade Universitária—Ilha do Fundão, Rio de Janeiro CEP 21941-596, Brazil

## Abstract

Macauba seed cake, a carbohydrate-rich biomass from seed mechanical oil extraction, is a promising feedstock for sustainable biorefinery applications. In this study, seed cake polysaccharides were characterized, and a mechanochemical pretreatment using planetary ball milling was applied as a chemical-free strategy to overcome biomass recalcitrance. The effects of dry grinding at different times (30, 60, 120, 180, and 240 min) were evaluated through ^13^C solid-state nuclear magnetic resonance, X-ray diffraction, and mid-infrared FTIR spectroscopy. The characterization of macauba seed cake confirmed it to be a rich source of d-mannose (44%). Solid-state ^13^C CPMAS NMR proved to be highly sensitive for monitoring mannan I polymorphs and revealed progressive amorphization during the pretreatment. Ball milling significantly reduced mannan crystallinity (from 26.6% in untreated biomass to 1.2% after 120 min and 2.4% after 180 min of pretreatment), promoted polymorphic transformations, and induced structural modifications in the polysaccharide functional groups. These structural changes improved polysaccharide bioavailability, leading to improved enzymatic hydrolysis by mannanases produced from *Aspergillus niger*. Saccharification yields, using 20 IU of mannanases per gram of dry biomass, reached up to 76.8% mannose release after 120 min of milling, confirming the strong correlation among crystallinity reduction, amorphization of mannan I, and enzymatic digestibility. Overall, macauba seed cake was validated as a promising feedstock for mannose recovery and other reducing sugars. To our knowledge, this is the first report of mannose production from macauba seed cake via ball milling and enzymatic hydrolysis and the first to demonstrate solid-state NMR as a tool to track amorphization in mannan-rich biomasses.

## Graphical abstract

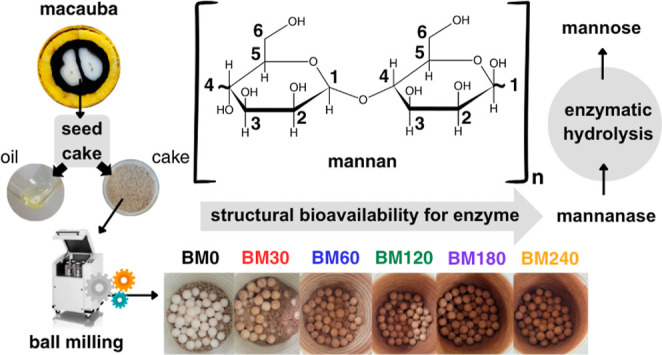

## Introduction

Carbohydrates are biomolecules with broad industrial applications, ranging from biofuels,^1^ nanomaterials,^2^ adhesives,^3^ food,^4^ pharmaceuticals,^5^ environmental processes,^6^ and other sectors of the chemical industry. The global carbohydrate commodity market is linked to the agricultural sector, including grains, timber, cotton, soybean meal, groundnuts, as well as rice, corn, sorghum, barley, and wheat.^7^ Another important source of carbohydrates is industrial residues or coproducts generated during the extraction of vegetable oils from oleaginous raw materials. Major commodities such as palm oil, palm kernel oil, soybean oil, rapeseed oil, sunflower oil, groundnut oil, and coconut oil accounted for around 172 Mt of oil in 2022,^8^ generating thousands of tons of biomass residues. Moreover, new oilseed crops with high potential such as macauba (*Acrocomia aculeata*) are emerging.

*A. aculeata* is a perennial, heliophytic, monoecious, allogamous species native to tropical regions of the Americas, with wide distribution in Brazil. Its main ecotypes are sclerocarpa, totai, and intumescens.^9^ The sclerocarpa ecotype produces the largest fruits and exhibits the most promising mesocarps for oil production. Macauba fruits are composed approximately of epicarp or shell (20%), mesocarp (40%), endocarp (33%), and seed (7%) as reported by Ciconini et al.,^10^ Colombo et al.,^11^ and Sorita et al.^12^ Interest in macauba has increased due to its high oil yield from pulp (0.6–3 tons per hectare) and seed (0.4–0.75 tons per hectare),^9,13^ positioning it as a potential alternative to the African oil palm (*Elaeis guineenses*), while also enabling the production of additional platform molecules from residual biomass.^14^

Processing the macauba fruit to obtain oils generates a significant amount of coproducts (20 tons per hectare),^12^ including pulp and seed cakes, epicarp, and endocarp (seed casing). Despite being rich in fiber, carbohydrates, proteins, and other biomolecules, these coproducts are little characterized and used mainly as animal feed, underscoring the need for studies to explore their potential in bioeconomy.^12,15^ Thus, although macauba lipids are widely studied, comprehensive knowledge of their carbohydrate composition and structure in fruits and coproducts is still lacking.

The seed is considered the most noble fraction of the fruit as it is rich in high-quality oil (48.5–55.9% dry matter), mainly lauric acid, suitable for cosmetic and food applications, as well as proteins (14.5–30.1% dry matter) and insoluble fibers (13.1–27.2% dry matter), as reported by Coimbra et al.,^16^ Hiane et al.,^17^ Lescano et al.^18^ and Belén-Camacho et al.^19^ Seed oil extraction generates a large amount of seed cake.^20^ For example, in a native plant production scenario in Brazil, seed cake generation is estimated at approximately 1–1.3 tons per hectare/year.^9^ This quantity represents more than 70% of the total seed. Regarding carbohydrates in seed or seed cakes, mannan is expected as storage carbohydrates, as observed in studies of other *Arecaceae* family fruit seeds such as *Phoenix dactylifera*,^21,22^ *Euterpe edulis Martius,* ^23^ and *Phytelephas macrocarpa*.^24^ The presence of endo-β-mannanases in *A. aculeata* seeds indirectly supports this assumption.^25^ It is known that seed maturation is influenced by embryo development and the action of endo-β-mannanases, which regulates mannan reserves in *Arecaceae*.^23,26^ The carbohydrate type, structure, and concentration change throughout fruit development. In addition to the mannans, which provide structural resistance to the cell wall, the seed may also contain cellulose, proteins, glycoproteins, proteoglycans, and minerals, as well as phenolic compounds in the tegument.^27–29^

Mannans are composed predominantly of β-(1 → 4)-linked d-mannopyranosyl residues.^30^ Mannan is classified into four subfamilies based on structural features: linear mannan, glucomannan, galactomannan, and galactoglucomannan.^31^ Linear mannans are water-insoluble and confer mechanical strength and crystallinity to seeds’ cell wall. The polymorphs mannan I and II, often co-occurring, differs in molecular weight and crystalline morphology: mannan I is anhydrous with lower molecular weight, while mannan II is more hydrated and less crystalline and has a microfibrillar morphology.^31^ Linear mannans consist mainly of d-mannose residues with less than 5% of galactose.^32,33^ Glucomannans consist of d-mannose and d-glucose residues intercalated. Galactomannan main chain contains only d-mannose and has d-galactose branches linked by α-1,6 bonds. Galactoglucomannan has a main structure like glucomannan but contains d-galactose branches. Also acetylated galactoglucomannans has a main structure like galactoglucomannan more *O*-acetyl groups.^30,33,34^

Mannans possess a significant degree of crystallinity that imparts hardness to seeds and protects them from mechanical damage.^35^ However, this crystallinity limits their utilization in biorefinery applications, as structural recalcitrance reduces enzymatic accessibility to obtain carbohydrates. Pretreatments that disrupt crystalline domains are therefore required to obtain monosaccharides and oligosaccharides from mannan-rich residues. Among available strategies, mechanochemical methods, particularly ball milling, are highly effective in promoting carbohydrate amorphization.^36–39^ Mechanochemistry can also induce partial polysaccharide degradation, although the threshold between amorphization and degradation remains unclear. The precise determination of amorphization by X-ray diffraction (XRD) requires complementation with other analyses, such as solid-state NMR. Previous studies demonstrated that solid-state NMR provided superior resolution for polysaccharide identification and monitoring of structural deconstruction during physical and enzymatic processing^36^ and during seed maturation.^23^

In summary, the limited knowledge of macauba carbohydrates constrains the valorization of its coproducts into higher-value bioproducts. Studies on macauba coproducts remain scarce, and the application of mechanochemical pretreatment followed by enzymatic hydrolysis is still unexplored for this biomass. The initial hypothesis considers that the seed cake of macauba palm is mainly composed of highly crystalline linear mannan-type polysaccharides, which limit the enzymatic accessibility. Furthermore, it is assumed that the mechanochemical pretreatment by planetary ball milling reduces mannan crystallinity and promotes amorphization, thus increasing the efficiency of enzymatic hydrolysis. The degree of amorphization is specifically monitored by ^13^C CPMAS solid-state NMR and correlated to mannose release, establishing a quantitative relationship between crystal rupture and carbohydrate accessibility. Therefore, the present study aims to (i) confirm the presence of linear mannan generally found in palm seeds, (ii) evaluate the structural changes in mannan polymorphism, crystallinity, and functional groups during ball milling, and (iii) elucidate the connection among mannan crystallinity, mechanochemical amorphization, and mannose release after enzymatic hydrolysis using fungal mannanases. The analyses employed included HPLC, ^1^H and ^13^C solution NMR, Fourier transform infrared (FTIR), XRD, and ^13^C solid-state NMR.

## Experimental Section

### Materials

The macauba seed cake was donated by the company INOCAS (Minas Gerais, Brazil) for the 2022 harvest. Sulfuric acid (Synth, 98%, Brazil), *n*-hexane (ACMA, 100%, Brazil), ethanol (Montenegro, 92.8%, Brazil), citric acid (Isofar, 99.5%, Brazil), sodium citrate (Isofar, 99%, Brazil), 3-(trimethylsilyl)-1-propanesulfonic acid sodium salt (DSS, Aldrich, 98%, Germany), and NMR grade deuterium oxide (D_2_O, Aldrich, 99%, Germany) were used as received. Ivory nut standard (mannan I), Avicel cellulose standard (microcrystalline), LBG standard (locust bean gum from *Ceratonia siliqua* seeds), d-(+)-mannose, d-(+)-glucose, d-(−)-fructose, d-xylose, d-(+)-galactose, d-(−)-arabinose, sucrose, and d-(+)-cellobiose standard with a purity of 99% were obtained from Merck KGaA (Germany), Sigma-Aldrich (Germany), and Start BioScience (USA).

### Methods

#### Preparation of Biomass for Processing

The biomass was milled in a cutting mill model Pulverissette 19 (Fritsch, Germany), equipped with a 1.5 mm sieve for particle size reduction, and dried in open air to less than 10% moisture. Dried samples (6.0 g, in dry weight) underwent solvent extraction in a Soxhlet apparatus, using *n*-hexane for lipid removal, and were stored in a desiccator (samples named BM0).

#### Ball Milling Pretreatment

Ball milling was performed in a planetary ball mill model PM 400 (Retsch, Haan, Germany) with 250 mL zirconium oxide vessels, each containing 50 zirconia balls (average diameter of 9.6 mm). Six grams (6.0 g) of BM0, with moisture below 10%, was dry-ground in a ball mill at 400 rpm in 10 min cycles with pauses (from 0 to 240 min) in triplicate, with a ball-to-biomass ratio of 24:1. The total rotating mass per jar (jar + lid + balls + biomass) was 4.728 kg (18.912 kg for four jars).

The energy calculation estimate (kWh/kg) was based on the manufacturer’s fixed mechanical demand of 1.5 kW, multiplied by the grinding times, and then divided by the quantity of biomass in each batch (in kg). The same can be done considering the manufacturer’s energy demand of 2.2 kW.

Samples were identified according to the ball milling time, namely, BM0 (0 min), BM30 (30 min), BM60 (60 min), BM120 (120 min), BM180 (180 min), and BM240 (240 min) according to Figure S1 (Supporting Information). Those grinding conditions were adapted from Panaro et al.^40^ for macauba seed cake at the Bioethanol Laboratory (IQ/UFRJ). All samples were analyzed by FTIR, XRD, and solid-state nuclear magnetic resonance.

#### Enzyme Production

The wild strain used was *Aspergillus niger* (code 1234) from the Amazon Cultivation Collection (CFAM—Fiocruz), originally isolated from drinking water in the Serra Baixa community of Iranduba (Amazonas, Brazil). The strain was propagated on potato dextrose agar (PDA, Sigma-Aldrich, Germany) at 27 °C for 7 days in an incubator (Incucell 111, MMM Group, Germany). Spores were suspended in 0.9% NaCl and centrifuged at 9000 rpm for 15 min, and the supernatant was discarded. The pellet was resuspended in 20% glycerol to obtain 10^8^ spores·mL^–1^, aliquoted into cryogenic vials, and stored at −18 °C.

Submerged fermentation was carried out with 300 mL of medium in 1 L Erlenmeyer flasks, incubated in a shaker (Innova 44R, New Brunswick, USA) at 200 rpm and 30 °C. The medium contained (g/L) 1.2 NaNO_3_, 3.0 KH_2_PO_4_, 6.0 K_2_HPO_4_, 0.05 CaCl_2_·2H_2_O, 0.2 MgSO_4_·7H_2_O, 0.002 FeSO_4_·7H_2_O, 0.016 CoCl_2_·6H_2_O, 0.005 MnSO_4_·4H_2_O, 0.0014 ZnSO_4_·7H_2_O, 12 yeast extract, and 30 locust bean gum (LBG). After sterilization, the medium was inoculated with 1% (v/v) of the spore suspension.

Mannanase activity was assayed using LBG (0.5% w/v) in 50 mM sodium citrate buffer (pH 4.8) as a substrate.^41^ After 10 min of reaction, reducing sugars were quantified according to Teixeira et al.^42^ One unit (U) of enzymatic activity was defined as the amount of enzyme releasing 1 μmol of d-mannose per min at 50 °C.

#### Enzymatic Hydrolysis

Enzymatic saccharifications were carried out in triplicate at 50 °C, 200 rpm, for up to 72 h using 10% solids (m/v) and a customized *A. niger* (code 1234) mannanase cocktail (20 IU per gram of dry biomass). A volume of 25 mL of reaction medium, consisting of 500 mM sodium acetate buffer (pH 4.8) and the enzyme cocktail, was added to the hydrolysis reactors. Both untreated and pretreated samples were hydrolyzed, and carbohydrates released were quantified using the DNS reagent (3,5-dinitrosalicylic acid) and identified as monosaccharides with a high-performance liquid chromatography (HPLC) system and by ^1^H and ^13^C solution nuclear magnetic resonance analysis.

### Characterization

#### Biomass Composition

Chemical characterization of the macauba seed cake followed the analytical protocols of the National Renewable Energy Laboratory (NREL, USA) in the determination of moisture (NREL/TP-510-42,621), ash (NREL/TP-510-42,622), extractives (adapted from NREL/TP-510-42,619), structural carbohydrate, and lignin (NREL/TP-510-42,618) following Sluiter et al.^43^ It was only possible to determine the insoluble lignin and proteins (as insoluble solids). The lipid content was determined by discounting the cartridge weights before and after *n*-hexane extraction on a dry basis. For biomass characterization, the removal of nonstructural materials (extractives) was performed using a two-step extraction process with ethanol (extractive sample named BM0-E) and water, each being used for 4 h. Structural monosaccharides were identified and quantified after acid hydrolysis (sample named BM0-AH) using an HPLC system and by ^1^H and ^13^C solution nuclear magnetic resonance analysis.

#### Dosage of Reducing Sugar by DNS Reagent

The DNS assay, adapted from Miller^44^ and Teixeira et al.,^42^ was used to construct the calibration curve and to determine the reducing sugars during the hydrolysis experiments and mannanase assays. Reactions containing 0.5 mL of the sample plus 0.5 mL of DNS were boiled for 5 min, followed by 6.5 mL of water. The absorbance was measured at 540 nm (Shimadzu UV-2600, Japan). All of the analyses were performed in triplicate.

#### High-Performance Liquid Chromatography

HPLC analyses were performed on an Ultimate 3000 system (Thermo Scientific, USA) with a refractive index detector, RI-101 (Shodex, Japan). Data acquisition and processing were controlled by the Chromeleon 7.1 software (Dionex Ltd., Canada). The column set comprised an ash removal precolumn (4.6 mm I.D. × 30 mm, BioRad, USA), Aminex Carbo P precolumn (4.6 mm I.D. × 30 mm, BioRad, USA), and Aminex HPX-87P analytical column (7.8 mm I.D. × 300 mm, BioRad, USA). The chromatographic conditions used were deionized water with a flow rate of 0.6 mL/min as the mobile phase, an injection volume of 10 μL, and a 30 min run time. Mannose yields were calculated using eq 1.

1 $$Y_{Man}\left(\%\right)=\frac{C_{Man.\left(\frac{V}{1000}\right)}}{M_{0}.B}\times 100$$

where *Y*_Man_ is mannose yield (%); *C*_Man_ is the mannose concentration after hydrolysis (g/L); V is reaction volume (mL); *M*_0_ is the theoretical mass percent of mannose (g); and *B* is dry biomass (g).

### ^1^H and ^13^C Solution Nuclear Magnetic Resonance Analysis

Liquid-state ^1^H and ^13^C NMR spectra were obtained using a Bruker Avance III 500 MHz (11.75 T) spectrometer operating at transmitter frequencies of 500.13 MHz (^1^H) and 125.77 MHz (^13^C), respectively. 1D ^1^H NMR spectra were obtained with zg30 pulse program and zgpr pulse program to water signal suppression with 65,536 of TD (time-domain), 1s recycle delay, 3.28s acquisition time, 16 scans, 20 ppm spectral width, and 0.30 Hz line broadening. 1D ^13^C NMR spectra were obtained with a zgpg30 pulse program with 65,536 TD, 2s recycle delay, 1.1s acquisition time, 512 scans, 236.6 ppm spectral width, and 1.0 Hz line broadening. Chemical shifts were expressed in parts per million (ppm). All samples were dissolved in the D_2_O solvent (deuterium oxide). Approximately 0.6 mL was transferred to a 5 mm NMR tube. The internal reference for chemical shifts was DSS (3-(trimethylsilyl)-1-propanesulfonic acid sodium salt).

### FTIR Analysis

FTIR analyses were performed in duplicate using the KBr-disk method on a Nicolet 6700 spectrophotometer (Thermo Scientific Corporation), mid-infrared region, 16 scans, DTGS-KBr detector, KBr beamsplitter, and 4 cm^–1^ resolution. Approximately 2 mg of the powdered sample was mixed and macerated with approximately 98 mg of KBr, and then the mixture was pressed to form a disk. The spectra were obtained in the frequency range of 400–4000 cm^–1^.

### XRD Analysis

Crystallinity was monitored in duplicate using an Ultima IV standard X-ray diffractometer (Rigaku, Japan) with CuKα radiation and a monochromator filter of Ni (wavelength of 1.5406 Å). The operating voltage was 40 kV, and a current of 20 mA was used in continuous scanning. The primary optics are based on copper K-α mirrors and 1/2° slits. The secondary optics are based on K-β filters with a receiving detector and a slit of 10 mm. The test samples were scanned within a 2θ angle range from 5° to 50°, K-beta filter method with a step (degree) of 0.02, and a speed duration time (degree/min) 10.0000. The crystallinity index value was calculated according to the adapted Ruland–Vonk method^45,46^ and using the mannan standard as a reference. The methodologies consisted of calculating the mannan crystalline phases considering five main signals in the 2θ angle range of 14.6–26° and the total area considering the peak at 16° as a reference. The degrees of crystallinity were calculated comparatively as the ratio of the area corresponding to the crystalline region to the total area, and as the ratio of the crystalline areas of all samples relative to the mannan standard. In all calculations, the regions below the baseline were subtracted.

### Solid-State Nuclear Magnetic Resonance Analysis

Solid-state ^13^C NMR spectra were obtained using a Bruker Avance III 400 MHz (9.4T) spectrometer operating at a transmitter frequency of 100.65 MHz, with a 4 mm multinuclear double channel CPMAS probe, cross-polarization magic angle spinning (CPMAS) pulse sequence, 10 kHz MAS rate, 0.5 ms contact time (optimized), 5s recycle delay, and 1000 scans. Samples of approximately 60 mg were transferred to 4 mm ZrO_2_ rotors stopped with Kel-F caps and analyzed at room temperature. The secondary standard reference was alpha-glycine (C=O at 176.03 ppm), and the primary standard reference was tetramethylsilane (BP, 27 °C) (0.0 ppm). Data processing was performed using TopSpin software, version 4.1.4, in the 200-0 ppm working window with automatic baseline and manual phase adjustments. Before the Fourier transformation, the free induction decay was multiplied by an exponential function of 30 Hz to minimize baseline noise. All calculations were performed using the width at half-height (Δ*ν*1/2, Hz) of the spectral signals. Ivory nut (mannan I) and Avicel cellulose (microcrystalline) were also used as analytical standards.

## Results and Discussion

### Biomass Compositional Analysis of Macauba Seed Cake

The composition of the macauba seed cake (sample BM0), performed using the standard NREL method, is listed in Table 1, and carbohydrate determination by HPLC, after acid hydrolysis (sample BM0-AH), is shown in Figure S2 (Supporting Information). Significant levels of structural carbohydrates, with d-mannose as the major component (44.0%), followed by d-glucose (8.6%), d-galactose (2.6%), and d-xylose (1.5%), and traces of d-arabinose (0.2%), were detected. Confirmatory analyses by solution NMR are shown in Figures S3 and S4 and Table S1 (Supporting Information). Insoluble solids (16.1%) represent additional potential bioresources, available alongside lignin and proteins, as well as soluble carbohydrates and lipids, collectively represented by extractives (33.0%), of which 23.5% were lipids extractable in *n*-hexane. The chemical composition revealed ash contents of 4.2% (inorganic compounds), similar to the value of 3.52% reported by Andrade et al.,^47^ and lipid contents of 23.5%, compared to 28.31% in the same study, despite methodological differences.

**Table 1:** Chemical Composition of the Macauba Seed Cake (Sample BM0) According to the NREL Protocols

Parameter	compositional (%)^a^
Moisture	7.6 ± 0.3
total ash	4.2 ± 1.2
Lipid	23.5 ± 2.5
extractives	33.0 ± 8.5
insoluble solids^b^	16.1 ± 1.4
mannose	44.0 ± 3.1
glucose	8.6 ± 0.7
galactose	2.6 ± 0.2
xylose	1.5 ± 0.1
arabinose	0.2 ± 0.07

a Data are presented as mean ± standard deviation of triplicate experiments.

b Excluding extractives.

The analysis indicated a high d-mannose content, suggesting a direct relationship with structural carbohydrates and the potential of the biomass to meet the market demand for carbohydrates. The predominance of d-mannose indicates that the seed cake is mainly composed of mannan polysaccharides, accounting for approximately 39.6% of its composition. This value is comparable to those reported for other palm species, such as 30–35% in *Elaeis guineensis*,^48^ and lower than the 52.46% in *Euterpe oleracea* ^49^ and 90% in *E. edulis Martius*.^23^ However, these data alone were insufficient to determine the specific type of mannans. Additional investigation was required to clarify the role of carbohydrates within the biomass matrix, as d-glucose and d-galactose may occur in different structural contexts.

Timell^32^ reported a Man/Glu/Gal percent ratio of 43:5:1 for the linear ivory nut mannan, and Moreira et al.^33^ estimated less than 5% of galactose. In contrast, galactomannans have been reported with ratios of 2–4:0:1 ^33^, 1:0:2 ^50^, and 2:0:1;^34^ glucomannan with 3:1:0 ^33^ and 2:1:0;^34^ and galactoglucomannan with 3:1:1.^33,34^ In the present work, macauba seed cake showed a ratio of 44:8:2 (or 22:4:1), indicative of a limited structure, similar to the linear ivory nut mannan structure. It is important to emphasize that carbohydrate structure changes depending on fruit maturity and postharvest handling,^35,51^ and less-branched structures are typical of mature fruits.

Regarding soluble carbohydrates extractable in ethanol (sample BM0-E), sucrose was the predominant sugar, followed by d-glucose and d-fructose, as shown by ^1^H and ^13^C solution NMR (Figure S5 and Tables S1 and S2, Supporting Information).

### Analysis of Enzymatic Hydrolysates by HPLC and DNS Reagent

The HPLC chromatograms of the enzymatic hydrolysates from macauba seed cake (BM0) and pretreated samples are shown in Figure S6 (Supporting Information). It should be noted that the d-mannose concentration measured by HPLC (retention time of 15.4 min) may be affected by coelution with d-fructose in the column used. Similarly, for the enzyme-free control samples, sucrose (or d-cellobiose) was released into the reaction medium with a retention time of 9.8 min after 60 min of pretreatment.

The coelution of sucrose with d-cellobiose and of d-mannose with d-fructose limited the accurate detection of these sugars using conventional chromatographic columns for sugars in HPLC. Different columns and chromatographic methods would be required to detect these sugars separately; therefore, complementary analytical techniques were essential to identify, differentiate, and quantify these sugars.

Hydrolysates from the milled samples were compared with the controls without an enzyme (only in sodium citrate buffer solution, pH 4.8). The chromatograms displayed intense peaks at 15.4 min, corresponding to d-mannose (with coeluting fructose), from 60 min onward. These results suggest that mannanase enzymes gained access to the biomass structures after 60 min of ball milling pretreatment. This finding is consistent with reducing sugar analysis of the same samples (Figures S7 and S8, Supporting Information). Furthermore, the peaks at a retention time of 9.8 min suggest the presence of sucrose or d-cellobiose after 60 min of pretreatment. However, the presence of d-cellobiose was not confirmed by ^1^H and ^13^C solution NMR (Figure S9, Supporting Information).

Table 2 shows the average concentration of reducing sugars, d-mannose, and d-glucose after 72 h of enzymatic hydrolysis, along with mannose yields (with or without fructose). From a processing standpoint, 60 min of ball milling is sufficient to enhance the accessibility of biomass to enzymatic attack, from 3.6% to 60.6% d-mannose yield. However, 120 min of pretreatment resulted in a better release of monosaccharides, with yields reaching 76.8%. The d-glucose concentration showed a similar behavior to the d-mannose concentration.

**Table 2:** Results of Reducing Sugar, Glucose, and Mannose Concentrations, as Well as Mannose Yields (%) with and without Fructose, from the Enzymatic Hydrolysate Samples Obtained at Different Ball Milling Times

sample	reducing sugar concentration	glucose concentration	mannose concentration			
	mM	g/L	g/L^a^	g/L^b^	yield, %^a^	yield, %^b^
BM0	1.1 ± 0.05	0.9 ± 1.7	1.6 ± 2.8	1.6	3.8	3.6
BM30	1.3 ± 0.05	4.1 ± 0.8	5.5 ± 1.9	5.2	12.5	11.9
BM60	6.6 ± 0.4	6.3 ± 2.4	27.9 ± 2.0	26.5	63.8	60.6
BM120	9.5 ± 0.7	8.2 ± 1.9	35.3 ± 6.8	33.5	80.7	76.8
BM180	7.7 ± 0.05	8.7 ± 1.9	36.0 ± 9.2	34.2	82.4	78.4
BM240	8.8 ± 0.2	8.2 ± 3.2	33.9 ± 4.5	32.2	77.6	73.8

a Considering the presence of fructose.

b without fructose.

Figures S7 and S8 (Supporting Information) show the variation in reducing sugar concentration measured by the DNS method and d-mannose by HPLC, as well as the d-mannose yield in samples BM0 to BM240. Table S3 (Supporting Information) shows statistical analysis of the calibration curve for quantification of reducing sugars. To the best of our knowledge, this is the first report of d-mannose yield from the macauba seed cake process by ball milling and enzymatic hydrolysis.

### ^1^H and ^13^C Solution Nuclear Magnetic Resonance Analysis

^1^H NMR spectra of BM0 (Figure S10, Supporting Information) and the pretreated samples after enzymatic hydrolysis display a complex pattern, making it challenging to characterize the monosaccharides, as the presence of residual enzyme absorptions in the same region was also confirmed.

On the other hand, the presence of sucrose and its hydrolysis products (d-glucose and d-fructose) could be confirmed by the ^1^H and ^13^C solution NMR spectra (Figures S11 and S12 and Tables S4 and S5, Supporting Information). NMR data of references are shown in Figures S13 to S16, Supporting Information).

These findings confirmed the invertase activity of the custom enzymes used and the presence of the monosaccharides d-mannose, d-glucose, and d-fructose (Figure 1). The ability of the fungus *A. niger* to produce invertases (1,2-β-D-fructofuranosidase frutohidrolase, EC 3.2.1.26) is widely known,^52,53^ in addition to its notable potential for producing β-mannanases (1,4-β-D-mannan mannohydrolases—EC 3.2.1.78)^54,55^ and other carbohydrate-active enzymes.^56^ This enzymatic versatility explains the *A. niger* relevance for efficient carbohydrate degradation in industrial processes.

**Figure 1:**
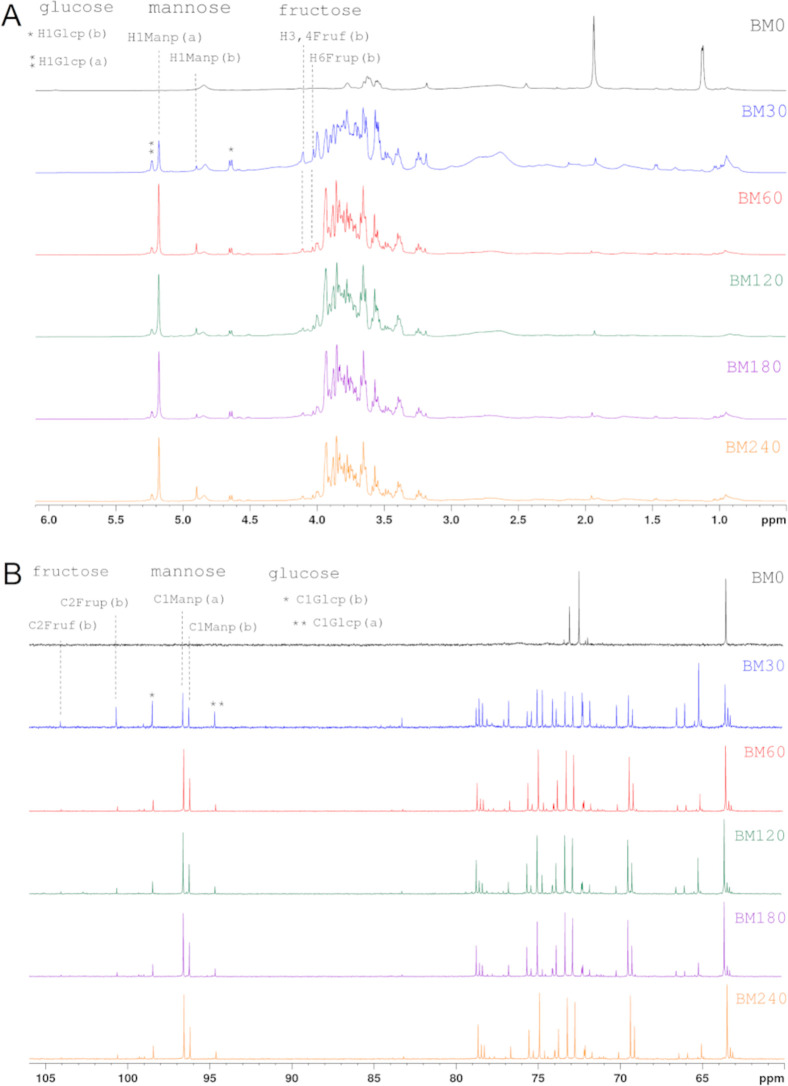
Solution NMR spectra obtained from samples after different milling times. (A) ^1^H (D_2_O, 500 MHz); (B) ^13^C (D_2_O, 125 MHz). (Man—mannose, Glc—glucose, Fru—fructose, p—pyranose, f—furanose, a—alpha, b—beta).

After sequential *n*-hexane and ethanol extraction (sample BM0-E), the most intense ^13^C NMR signals revealed the presence of sucrose, with characteristic resonances at 106.3 ppm (C2 of β-D-fructofuranose) and 94.8 ppm (C1 of α- *d* -glucopyranose). Additionally, lower-intensity signals were attributed to d-glucose (98.5 ppm, C1 of β- *d* -glucopyranose, and 94.8 ppm, C1 of α- *d* -glucopyranose) and d-fructose (104.1 ppm, C2 of β-D-fructofuranose, and 100.7 ppm, C2 of β-D-fructopyranose). These assignments were confirmed by ^1^H NMR analysis, such as sucrose (5.40 ppm, H1 of α- *d* -glucopyranose, and 3.67 ppm, H1 of β-D-fructofuranose), d-glucose (5.22 ppm, H1 of α- *d* -glucopyranose, and 4.64 ppm, H1 of β- *d* -glucopyranose), and d-fructose (3.99 ppm, H5 and 4.01 ppm, H6 of β-d-fructopyranose, and 4.10 ppm, H3 of β-d-fructofuranose).

The presence of sucrose signals indicates that it is the main extractable carbohydrate, and that upon enzymatic hydrolysis, it accounts for the small amounts of d-fructose found in high-mannose enzymatic hydrolysates. Although the spectra were not quantitative, assuming comparable nuclear Overhauser effects and the longitudinal relaxation times for carbons of the same type (e.g., OCH_2_ or OCH), the relative areas of C5 or C6 of d-mannose (piranosyl ring) and d-fructose (furanosyl ring) were integrated. These measurements suggested a d-fructose contribution in the range of 1–5%.

### FTIR Analysis

Figure 2 shows the FTIR spectra of macauba seed cake (BM0) compared to the ivory nut mannan and microcrystalline cellulose standards.

**Figure 2:**
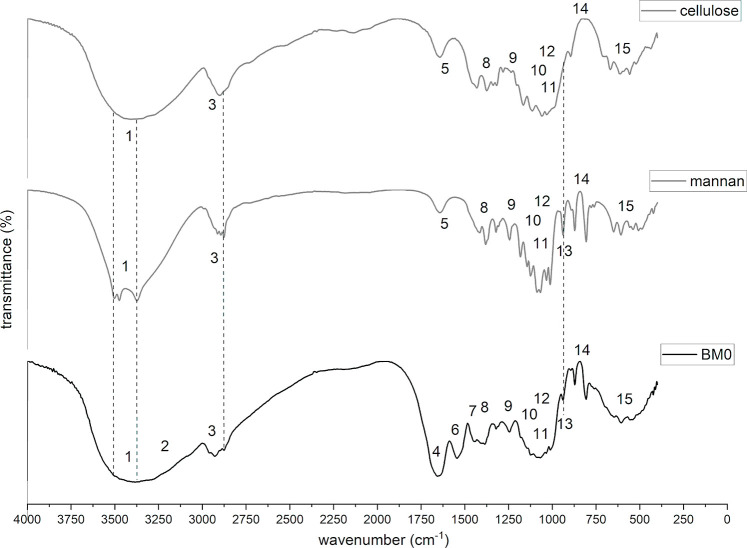
FTIR spectra of macauba seed cake (BM0), ivory nut mannan, and microcrystalline cellulose. Regions assigned as 1 to 15 are detailed in the text.

The BM0 spectrum exhibits absorption bands in the range of 3750–3000 cm^–1^ (regions 1 and 2), attributed mainly to O–H and N–H stretching vibrations involved in inter- and intramolecular hydrogen bonds. Stretching bands at 2929-2876 cm^–1^ correspond to C–H absorptions (region 3). These results were consistent with those of Hong et al.,^57^ which attribute greater importance to the 4000–2500 cm^–1^ region for polysaccharide analysis, where a strong and broad band between 3600 and 3000 cm^–1^ is associated with O–H stretching (abundant in polysaccharides), and moderate bands between 3000 and 2500 cm^–1^ are attributed to symmetric and asymmetric stretching of C–H and CH_2_. In the BM0 spectrum, absorptions in the range between 3350 and 3180 cm^–1^ are associated with N–H bond stretching.^58,59^

The proteins present in BM0 can be recognized mainly by the amide carbonyl bands (amide I region) with absorption of primary amides at 1657 cm^–1^ (region 4), in addition to a second band (region 6) at 1544 cm^–1^ (amide II region) that includes the bands associated with N–H and C–N angular deformation interactions. In addition, there is a contribution from the C–N stretching at 1441 cm^–1^ (region 7) and amide-related bands near 600 cm^–1^ (complex region at 15), in agreement with Gomba et al.^60^ Similar protein-associated bands at 1650, 1550, and 1400 cm^–1^ were reported by Boulet et al.^61^ in polysaccharides. By contrast, bands at 1642 and 1643 cm^–1^ (region 5), observed in the cellulose and mannan standards, were suggestive of alcohol O–H bending, which, in the case of sample BM0, was not clearly identified due to the overlap with the region of the amide I band.

Alcohol groups may also be associated with the moderate-intensity bands at 1386 and 1321 cm^–1^ (region 8), related to angular deformations of the C–O–H consistent with mannan absorptions at 1381 and 1322 cm^–1^. The band at 1245 cm^–1^ (region 9) suggests stretching of the C–O–C bond, present in both the BM0 sample and in mannan at 1244 cm^–1^. Cellulose presented a different band.

Strong bands between 1205 and 1050 cm^–1^ are generated by stretching vibrations of the C–O bond in alcohols and between 1150 and 1085 cm^–1^ (or around 1125 cm^–1^) and by C–O–C bonds in ethers.^58,59,61^ In this region, eight signals were observed for the BM0 sample, including bands at 1182 and 1143 cm^–1^ (region 10) of asymmetric C–O–C stretching of ethers, at 1124, 1087, and 1069 cm^–1^ (region 11) of C–O stretching of alcohols, at 1034 and 1011 cm^–1^ (region 12) of C–O stretching of alcohols and ethers, and at 939 cm^–1^ (region 13) of C–O stretching of alcohols. Those bands were also observed in mannan I and not observed in cellulose, as seen by Calumby et al.^62^ In particular, the bands between 1124 and 1087 cm^–1^ can be attributed to C–O–H groups at C2 and C3 (secondary alcohols) of mannan, while the band at 939 cm^–1^ could correspond to stretching of the primary alcohol C_6_–O–H.^58^

Small bands between 1000 and 800 cm^–1^ in the “anomeric region” correspond to stretching of C–O–C ether groups and bending of C–H bonds of glycosidic linkages.^57,63–65^ In this region, three absorptions were observed in sample BM0, bands at 894, 871, and 807 cm^–1^ (region 14) that reinforce the similarities with mannan (at 894, 872, and 806 cm^–1^, respectively), corresponding to the symmetric stretching of the glycosidic bond. In particular, the band at 872–871 cm^–1^ was attributed to the ether bond of the six-membered ring and that at 807–806 cm^–1^ was attributed to the ether bond of the glycosidic region between C1 and C4 of mannan. Cellulose presented only one band at 895 cm^–1^. Table S6 (Supporting Information) shows the detailed assignments of the observed absorptions.

Figure 3 shows the FTIR spectra of the samples after ball-milling. The pretreatment primarily affected the alcohol functional groups within the mannan structure. The most significant effects of ball milling are related to the alcohol group, mainly in the C–O stretching band region at 939 cm^–1^, where a reduction in band intensity (hypochromic shift) was observed starting from BM30 to sample BM120, when a discrete hyperchromic effect emerged in samples BM180 and BM240. That observation may suggest that components other than mannan present in low amounts and observed become more visible at longer pretreatment times. Similarly, broadening of the bands between 3400 and 3000 cm^–1^ was observed starting at 60 min. O–H stretching vibrations of inter- and intramolecular hydrogen bonds appear in this frequency range.^58^ This indicates that the pretreatment changed the hydrogen bonding pattern of the BM0 sample relative to the mannan crystallinity and that new molecular interactions are possible in the biomass.

**Figure 3:**
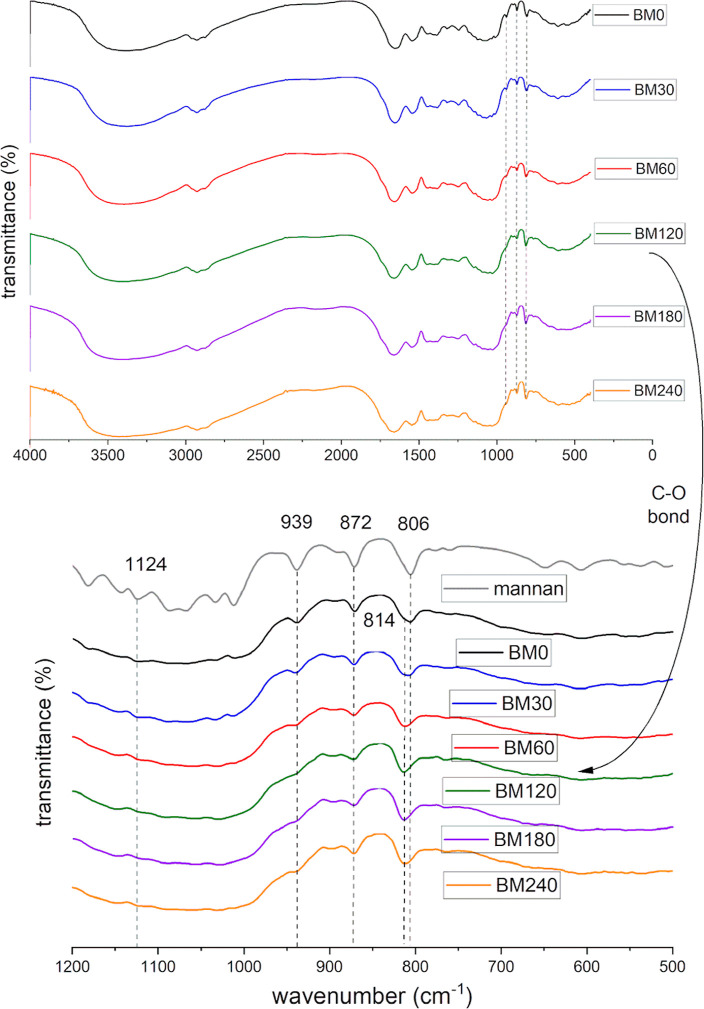
FTIR spectra obtained from samples after different milling times, compared to mannan.

On the other hand, the ether bonds corresponding to the absorption at 872 cm^–1^ were little affected by the pretreatment, which may indicate low mannan degradation up to 240 min, whereas a hypochromic effect was observed for the 806 cm^–1^ absorption, in parallel with a hyperchromic effect in the absorption at 814 cm^–1^, in samples BM180 and BM240. Those observations may suggest that components other than mannan, present in small amounts, become predominant with longer pretreatment times. No relevant changes were observed in the alkane and amide bands. The absorptions identified in the pretreated samples are summarized in Table S7 (Supporting Information).

### XRD Analysis

Figure 4 shows the diffractograms of the BM0 alongside the analytical standards of ivory nut mannan and microcrystalline cellulose. Seven diffraction peaks were identified, corresponding to the crystallographic planes 110, 111, 200, 210, 211, 120, and 004, at 2θ values of 16.1°, 18.4°, 20.2°, 23.7°, 25.2°, 26.7°, and 33°, respectively.

**Figure 4:**
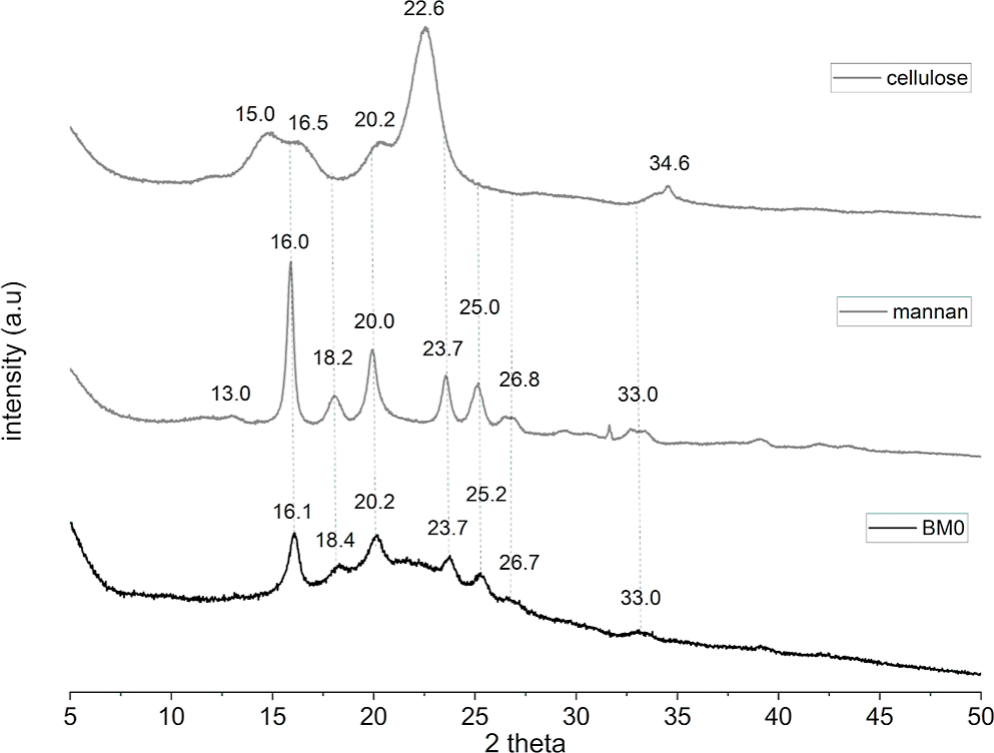
X-ray diffraction obtained from macauba seed cake (BM0), mannan, and microcrystalline cellulose.

The main diffraction peaks in the crystalline phase of the macauba seed cake were consistent with those observed for mannan. The identical peak positions suggest that the symmetries, space groups, and cell parameters were the same for both samples. However, differences in absolute and relative peak intensities suggest the presence of additional phases, other than mannan, and a slight change in the coordination of the atoms in the unit cell in sample BM0. In addition, there were slight changes in peak widths that correlated with crystal size. This behavior indicates the coexistence of more cell wall constituents commonly found in fruit seeds. Table S8 (Supporting Information) lists the XRD signals of both polysaccharides reported in the literature compared to those identified in macauba seed cake.

The mannan I allomorph, also known as linear mannan (poly-β-1,4-d-mannopyranose), typically found in higher plants, presents diffraction profiles as cited by Silva et al.,^24^ Grimaud et al.,^66^ and Gibril et al.,^67^ with a high degree of crystallinity such as that extracted from Ivory nut (*P. macrocarpa*). Recently, Mello et al.^23^ reported five diffraction peaks for crystalline mannan I at 2θ = 15.96°, 18.24°, 20.08°, 23.66°, and 25.18°. Slight variations may occur depending on the crystallographic environment, such as in the interaction with cellulose.^30^

Cellulose I (poly-β-1,4- *d* -glucopyranose) can also be present in seeds and exhibits some crystalline signals that coincide with those of mannan I. However, regardless of the morphology of cellulose I, the signals at 2θ = 16° and 22°^62^ are characteristic of this polysaccharide, with the most intense crystalline peak at 2θ = 22° and the region near 2θ = 20° a likely indicator of its presence.^68^ Murillo-Franco et al.^46^ suggest that açaí seeds consistently contain cellulose I and mannan I.

The intense signal identifies the possible presence of cellulose at 2θ = 16.8° and a lower signal at 2θ = 19.4° associated with the compacted hexagonal structures, in addition to the moderate signal at 2θ = 21.4° characteristic of the crystallographic plane (200) of cellulose I. The presence of mannan type I was recognized by the peaks at 2θ = 25° and 26.8°. However, variations may occur, as cited by Kanai et al.^69^ regarding the existence of three peaks for holocellulose generally found at 2θ = 16°, 20°, and 25°, corresponding to the main mannan I planes at 110, 200/102, and 211 ^70^. Cellulose peaks may also be prominent near 2θ = 11.9°, 21.4°, and 22.7°, suggesting the coexistence of cellulose I and cellulose II structures. Dutra et al.^71^ recall that the peak at 2θ = 11.9° in cellulose indicates the presence of amorphous regions, and the intense signal at 2θ = 21.5° signifies regions of high crystallinity that can reach up to 72%. In comparison to the values found in the literature, the crystallinity index can vary depending on the nature of the material and the calculation methods used.^72–74^

Figure 5 shows the XRD patterns after the ball milling. The main effect of pretreatment is a reduction in peak intensities with no significant shift in peak positions, indicating that symmetries, space groups, and unit cell parameters are largely maintained. The crystallinity index (CI) decreased progressively from 26.6% in BM0 to 20.7% at 30 min, 5.9% at 60 min, and 1.2% at 120 min (Table 3), indicating the near-complete amorphization of the main mannan phase.

**Figure 5:**
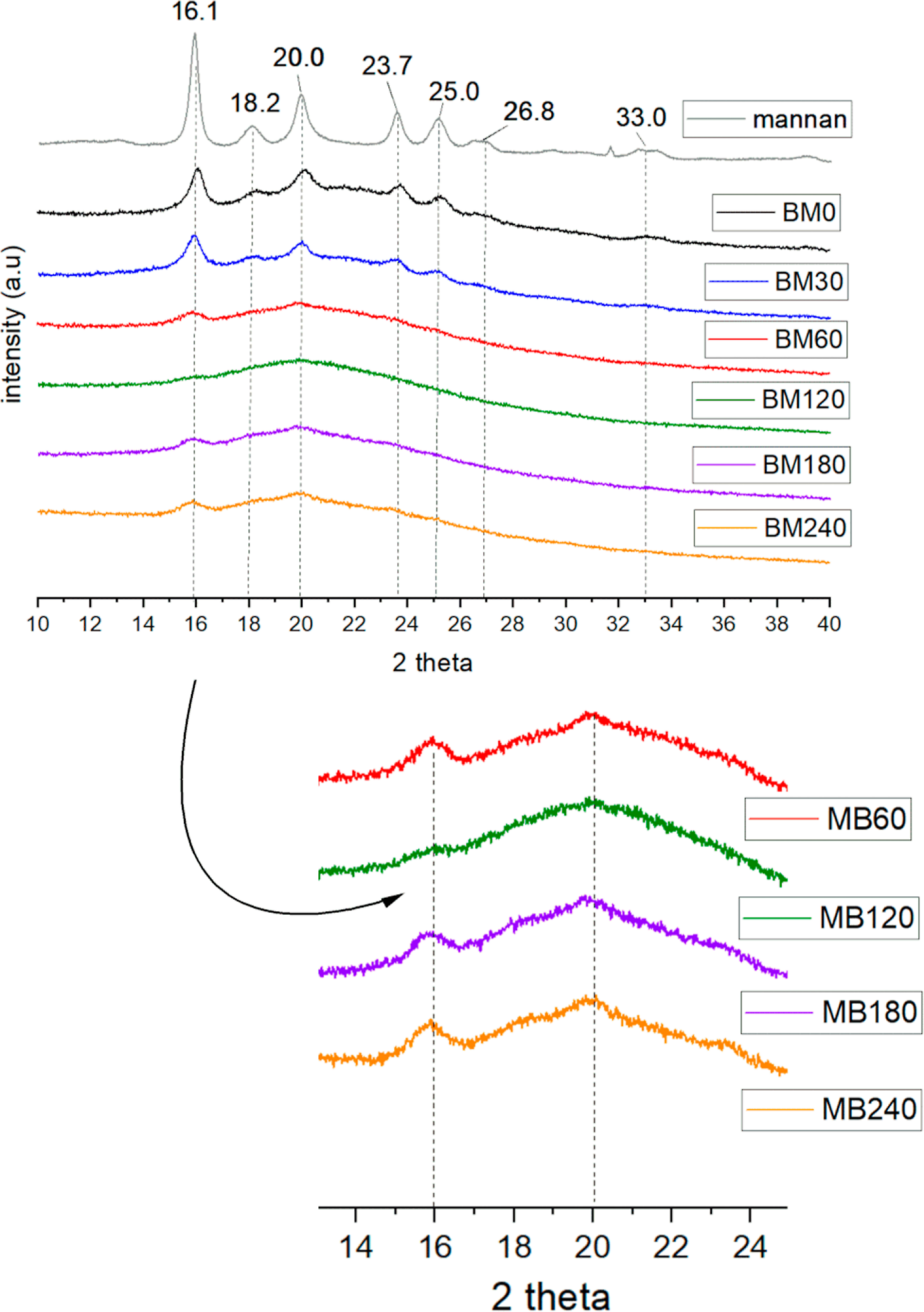
X-ray diffraction obtained for samples treated after different milling times.

**Table 3:** Crystallographic Data Obtained for Samples after Different Milling Times

samples	Miller indices/2θ °								CrI	CrI	reduction
	101	110^a^	111	200^a^	210	211	120	004/203	(%)^b^	(%)^c^	(%)^d^
mannan	-	15,9	18,1	19,9	23,6	25,2	26.6	33.0	-	76.3	-
BM0	-	16.1	18.4	20.2	23.7	25.2	26.7	33.1	26.6 ± 0.8	17.4 ± 0.5	-
BM30	-	15,9	17.9	20.0	23.6	25.2	-	-	20.7 ± 0.4	16.1 ± 0.5	22.2
BM60	-	15.9	0.0	19.8	0.0	0.0	-	-	5.9 ± 2.4	5.3 ± 1.4	77.8
BM120	-	16.8	0.0	19.9	0.0	0.0	-	-	1.2 ± 0.6	0.9 ± 0.4	95.5
BM180	-	15.8	0.0	19.8	0.0	0.0	-	-	2.4 ± 2.7	1.9 ± 2.0	91.0
BM240	-	15.9	0.0	20.1	0.0	0.0	-	-	4.8 ± 2.9	4.4 ± 3.0	82.0

a More intense diffraction peaks.

b Calculated from the relation between the crystalline area of samples and the mannan standard.

c Calculated from the relation between the crystalline area and total area of the diffractogram.

d Impact on Crystallinity Reduction.

This represents a significant modification in crystallinity, varying from 22.2% (BM30) to 95.5% (BM120), followed by slight reductions at longer milling times (91.0% for BM180 and 82.0% for BM240), which shows that planetary ball milling reduces biomass recalcitrance through structural disruption of the polysaccharide matrix. However, achieving a substantial impact on crystallinity requires energy input. Since both torque and rotational speed are not constant during milling, the energy demand varies throughout the process.

Nevertheless, it is possible to estimate energy consumption based on the specifications provided by the manufacturer Retsch. Considering a mechanical power of 1.5 kW, the estimated mechanical energy consumption was 31.3 kWh/kg (BM30), 62.5 kWh/kg (BM60), 125.0 kWh/kg (BM120), 187.5 kWh/kg (BM180), and 250.0 kWh/kg (BM240). For a rated energy consumption of 2.2 kW, the estimated values were 45.8 kWh/kg (BM30), 91.7 kWh/kg (BM60), 183.3 kWh/kg (BM120), 275.0 kWh/kg (BM180), and 366.7 kWh/kg (BM240). It should be emphasized that comparisons with other pretreatment technologies must be conducted with caution, ensuring consistency in units and careful verification of the calculation methodologies used for the energy accounting.

No less important is the fact that, at 180 min, CI remained similar (2.4%) but increased to 4.8% at 240 min due to the reappearance of the peak at 2θ = 16°. This may indicate that the biomass amorphization process was altered due to the presence of other phases in the material or the occurrence of mechanochemical reversibility.^75^

The signal at 2θ = 16° may be associated with the coincidence of the different phases present: the first phase of mannan, which lost crystallinity at 120 min, in addition to the possibility of greater evidence of the second unknown constituent phase, whose crystallinity may be more evident at longer pretreatment times.

The biomass amorphization process was thermodynamically favorable up to 120 min (CI = 1.2%) and extended to 180 min (CI = 2.4%) within the measurement error. The data suggest that mannan appears to be the main constituent in this biomass amorphization phase. However, after 120 min, the crystallinity of the second constituent appears to contribute to the cake crystallinity, resulting in an increase in crystallinity at 240 min (CI = 4.8%).

After 120 min, a different behavior of the mechanochemical process was observed. The hypothesis is that the second crystallographic contribution may be associated with the presence of amino acids.^76^ Due to the protein-rich nature of the macauba seed cake, combined with information from other analytical techniques, it is most likely that the second constituent is associated with the presence of protein crystals, especially glutamic acid, or glycoconjugates, such as l-asparagine to form *N*-glycosides, or l-serine and l-threonine to form O-glycosides.^77^

### ^13^C Solid-State Nuclear Magnetic Resonance Analysis

Solid-state ^13^C CPMAS spectra of BM0 and the standards (mannan, galactomannan, and cellulose) are shown in Figure 6. Polysaccharide spectral signals are dominant and evidence the presence of the homomannan-I polymorph (Figure 6A), through the chemical shifts (ppm): 101.6 (C1), 80.9 (C4), 75.8 (C5), 72.1 (C3), 69.8 (C2), and 61.9 (C6). Protein resonances may also appear in the region near 30 ppm, corresponding to C–H carbons typically found in aliphatic chains, as identified by Soares et al.^78^ Lessa et al.^79^ reported for the macauba protein isolate, a variety of amino acids, such as glutamate (Glu), arginine (Arg), aspartate (Asp), leucine (Leu), phenylalanine (Phe), valine (Val), lysine (Lys), isoleucine (Ile), methionine (Met), tyrosine (Tyr), and others, which intensify and broaden the spectral region between 46 and 10 ppm. The ^13^C chemical shifts are, for arginine: 157.7 ppm (C=O), 56.4–53.9 ppm (Cα), 41.1 ppm (Cδ), 28.7 ppm (Cβ), and 25.3 ppm (Cγ), and for glutamate: 178.2 ppm (Cδ), 56.4–53.9 ppm (Cα), 32–30.9 ppm (Cγ), and 28.7 ppm (Cβ).^80^

**Figure 6:**
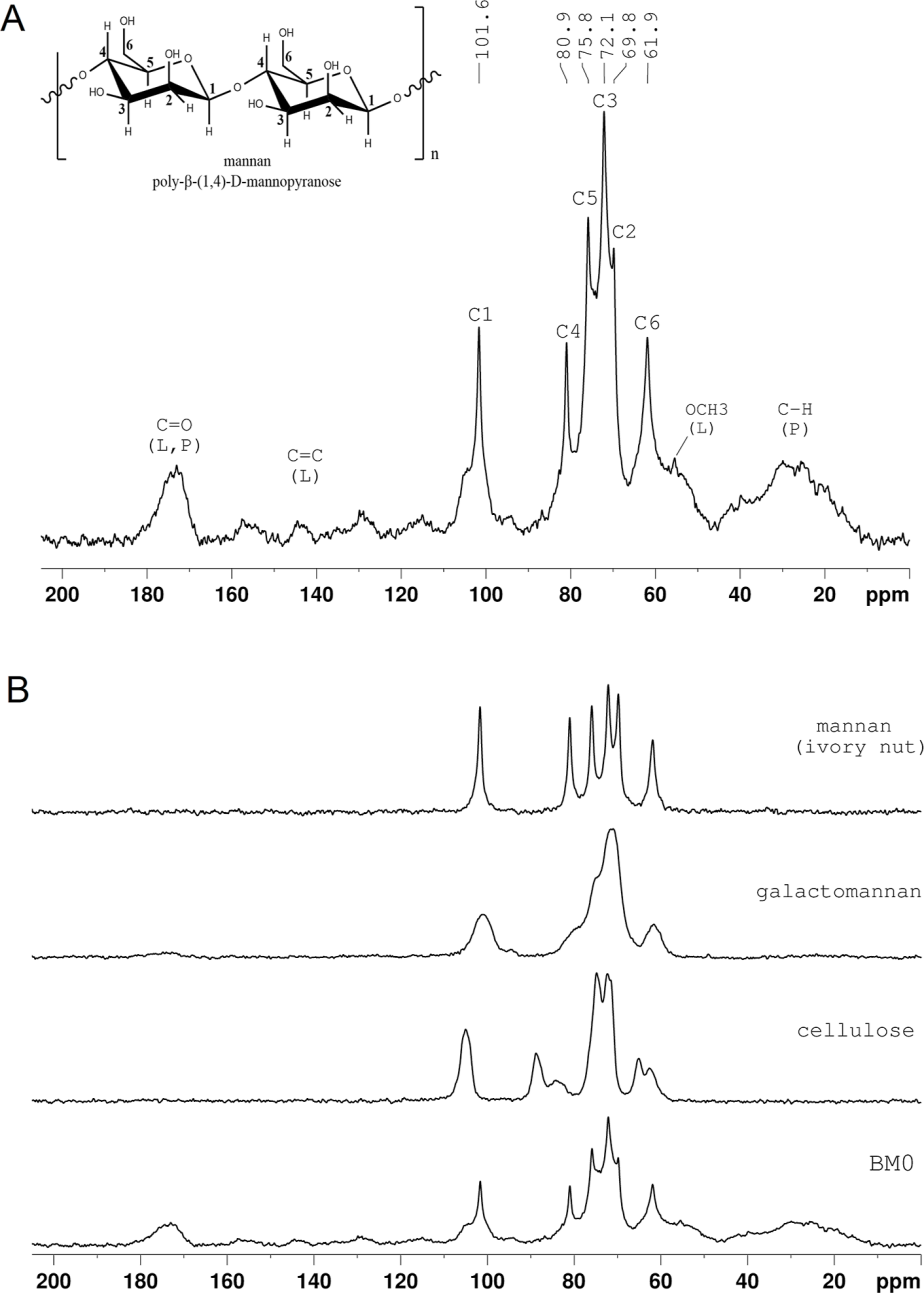
^13^C CPMAS NMR spectra (100 MHz): (A) sample BM0 of macauba seed cake (L: lignin, P: protein; C­(n): mannan carbon); (B) Ivory nut mannan, galactomannan, and cellulose standards.

Characteristic lignin resonances are also found: the methoxy group at 56 ppm and the aryl C of lower intensity between 160 and 110 ppm. These lignin signals are found centered at 157, 145, 129, and 115 ppm, suggestive of monomeric precursors of syringyl (S), guaiacyl (G), and *p*-hydroxyphenyl (H),^81–83^ and close to typical signals found in wood at 153 ppm (S), 145 ppm (G,S), and 115 ppm (H)^84,85^ in Figure 8B. More intense signals from the carbonyl (C=O) region at 173–174 ppm confirm the significant presence of proteins, in addition to lignin.

**Figure 7:**
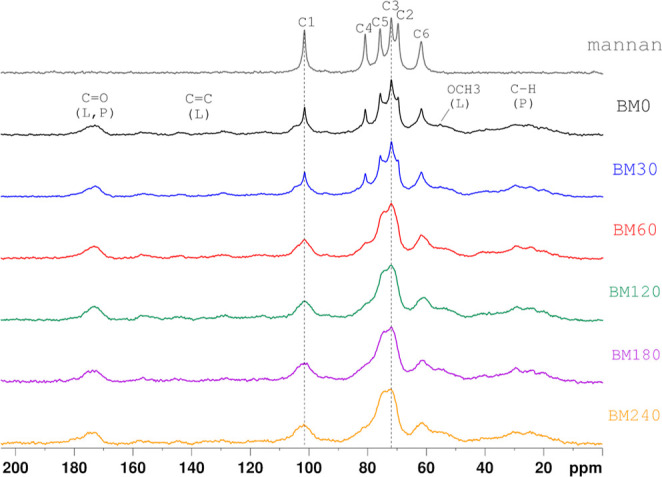
^13^C CPMAS NMR spectra (100 MHz) of macauba seed cake obtained after different milling times and comparison with the Ivory nut mannan standard (L-lignin; P- protein; C­(n): mannan carbon).

**Figure 8:**
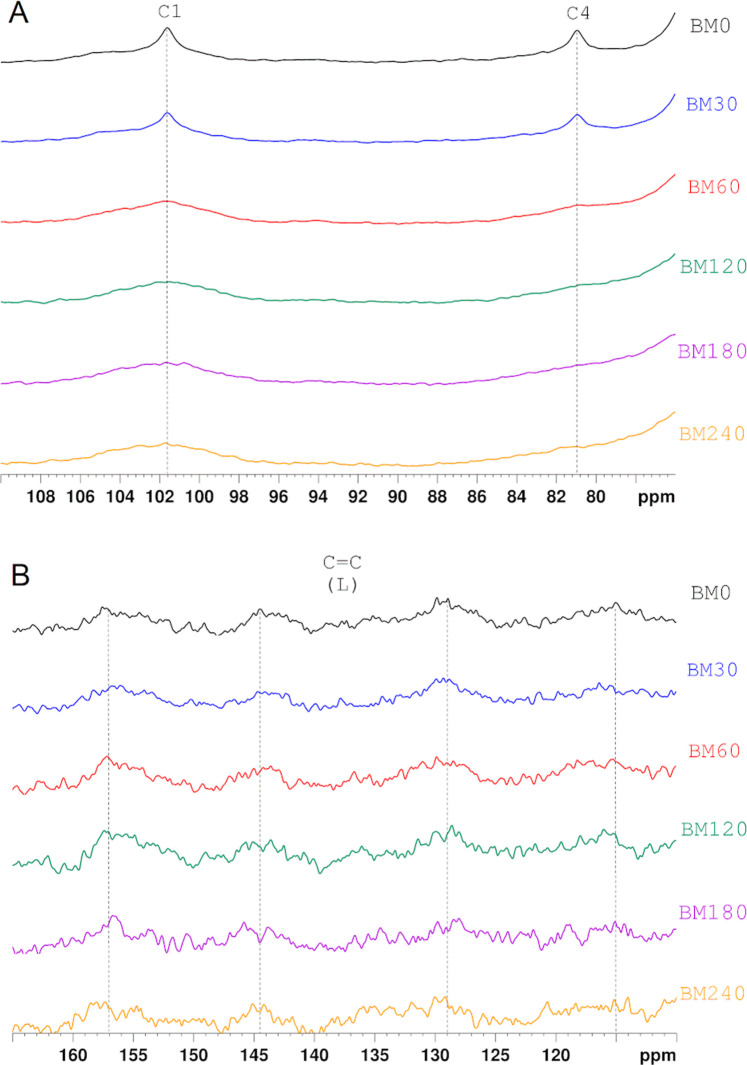
^13^C CPMAS NMR spectra (100 MHz): (A) expansion of the glycosidic bond region, 110–76 ppm; (B) aromatic lignin region.

Comparison with standards (Figure 6B) confirmed the predominance of linear mannan in the macauba seed cake obtained at the final stage of fruit ripening, with no structural evidence of galactomannan. The Ivory nut pattern corresponds to the six carbons of the β-(1 → 4)-d-mannosyl residues of crystalline mannan-I, such as 101.6 ppm (C1), 81.0 ppm (C4), 75.9 ppm (C5), 72.1 ppm (C3), 69.8 ppm (C2), and 61.9 ppm (C6), consistent with linear mannans reported in the literature.^86^ Mannan I polymorph was also reported in other palm seeds.^21,23,87^ Moura et al.^51^ report that the seed has a thicker cell wall due to the deposition of storage polysaccharides during the postgermination period, primarily hemicelluloses and mannan, which dominate the seed cell wall composition. The mannans found are of the “pure mannan” type and polysaccharides that contain less than 10% sugar residues other than mannose. No less important is the fact that the suspicion that the mannan II polymorph is present in the macauba seed cake is suggested by the observation of low-intensity spectral signals at 102.8 ppm (C1), 82.7 ppm (C4), 74.9 ppm (C5), 72.5 ppm (C3), 70.9 ppm (C2), and 62.9 ppm (C6), compared to the mannan II (*Codium fragile*) reported in the work of Marchessault et al.^87^ at 102.4 ppm (C1), 83 ppm (C4), 75 ppm (C5), 72.5 ppm (C3), 70.9 ppm (C2), and 62.9 ppm (C6), in the original form or after recrystallization in 100.5 ppm (C1), 76.8 ppm (C4), and 60.9 ppm (C6).^88^ However, mannan I remains the dominant polymorph.

Furthermore, it is evident that galactomannan is not the predominant polysaccharide in the macauba seed cake at this stage of maturation. Galactomannan is structurally more branched, with d-galactose residues, which impacts less-resolved spectral signals, as shown in Figure 6B. Corresponding galactomannan standard signals were observed at C1–101.3 ppm, C4–79 ppm, C5–75 ppm, C3–71.7 ppm, C2–71 ppm, and C6–61.6 ppm. Similarly, it is clear that cellulose is not confirmed as the main polysaccharide in the macauba seed cake. The cellulose standard presents typical signals at C1–104.9 ppm, C4c-88.8 ppm, C4a-84.5 ppm, C5–74.8 ppm, C3–72.3 ppm, C2–71.5 ppm, C6c-65 ppm, and C6a-62.5 ppm (c: crystalline; a: amorphous).

Regarding the possible other constituents superposed to the mannan I signals between 55 and 110 ppm, observed by the less intense signal in the region close to 105–104 ppm, it is suspected to be cellulose due to previous studies characterizing lignocellulosic biomass,^36^ where less intense cellulose I signals were observed in the region close to 105–104 ppm. Despite this, there is another suspicion that glycoconjugates may be part of the structure of macauba cake as biomarkers, as is the case in other plant seeds.^89^ However, the presence of these components could not be predicted, because the mannan signals overlap with those of other constituents.

The structural effects of ball milling are shown in the ^13^C CPMAS spectra (Figure 7), where the well-resolved linear mannan signals progressively decreased after 60 min. The spectral signal assignments for mannan, lignin (L), and protein (P) in untreated and pretreated macauba seed cake samples are visualized in Figure 7. The chemical shift assignments for the mannan carbons, as well as the tracking of the width-at-half-height (Δν1/2) parameter in untreated and pretreated macauba seed cake samples, are summarized in Table 4. Spectral changes indicate increasing structural disorder and amorphization. At 30 min, only minor alterations were observed, but from 60 min onward, amorphization became evident and persisted up to 240 min.

**Table 4:** Variation of Width at Half-Height (Δ*ν*1/2, Hz) Measured in the ^13^C CPMAS NMR Spectra of Macauba Seed Cake After Different Milling Times^a^

sample	Δ*ν*1/2 (Hz)					
	C1	C4	C5	C3	C2	C6
	101.7 ppm	81 ppm	76–74 ppm	72 ppm	71–70 ppm	62–61 ppm
BM0	171	220	222	344	96	453
BM30	196	584	299	395	84	360
BM60	492	263	710	227	70	425
BM120	585	311	633	410	267	593
BM180	626	568	739	249	134	552
BM240	662	184	626	398	317	558

a Not measured.

Beyond 120 min, the structure was largely disorganized and/or amorphous, creating conditions favorable for enzymatic attack. This was evident from the broadening of the glycosidic bond region (105–80 ppm, Figure 8A), where signal resolution markedly decreased, reflecting the loss of crystallinity of mannan I. Mechanochemical reorganization of the crystal lattice and the contribution of a secondary macromolecular constituent are suggested by the partial recovery of signal resolution after 180 min. However, the hypothesis of changes in the mechanochemical process^90^ of mannan deformation cannot be ruled out.

These results are consistent with the XRD and FTIR diffraction signals. Since the chemical shifts of the six carbons in the d-mannose backbone remained unchanged at all milling times, the effects of ball milling were best assessed through the width-at-half-height parameter (Δν1/2) after deconvolution of the mannan carbons. The width-at-half-height parameter (Δν1/2) of all mannan carbons changed throughout the pretreatment, with greater impacts observed after 60 min (Table 4). However, the impacts of ball milling are best observed in the spectral region corresponding to the glycosidic bond (Table 4 and Figure 8A), especially at carbon C1.

Figure 9 shows a correlation between the yield (%) and the concentration (g/L) of d-mannose (%) with the width-at-half-height (Δν1/2) parameter for the C1 carbon of mannan, measured in the ^13^C CPMAS NMR spectra for the BM0 and pretreated samples. The increase in Δν1/2 in the spectral signals expresses the loss of crystallinity and/or gain of amorphization of mannan. This parameter can be correlated with the enzymatic conversion of mannan, so that the increase in the yield of mannose release occurs from BM0 (3.6%) to BM60 (60.6%), reaching higher percentages in BM120 (76.8%), statistically similar to BM180 (78.4%), and decreasing in BM240 (73.8%). This also means an increase in concentration from BM0 (1.6 g/L) to BM60 (26.5 g/L), reaching BM120 (33.5 g/L), BM180 (34.2 g/L), and BM240 (32.2 g/L).

**Figure 9:**
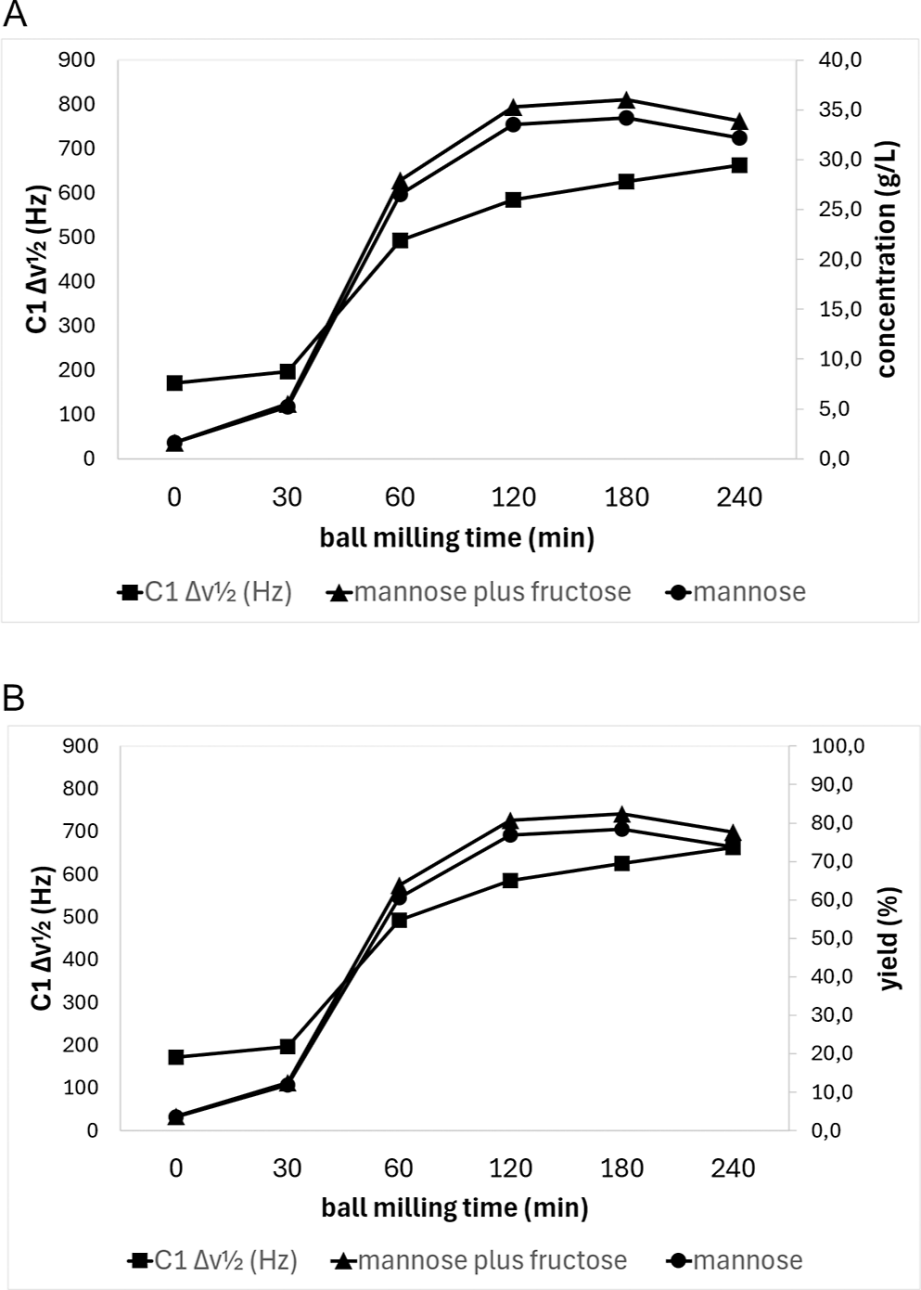
Correlation between the C1 half-height parameter (Δν1/2) measured by ^13^C CPMAS NMR spectra of samples BM0, BM30, BM60, BM120, BM180, and BM240 with (A) the mannose concentration in g/L; and (B) the mannose yield in %.

Another key point is the structural explanation for why the 120 min sample stands out as the best grinding condition studied. The main purpose of pretreatment is to increase the amorphous phase of mannan rather than to degrade it. The amorphization process is associated with the disruption of intermolecular hydrogen bonds, as confirmed by infrared bands, without affecting glycosidic bonds. These results are consistent with the ^13^C CPMAS NMR spectra. From 60 min onward, signal intensity decreases equimolarly, although a residual C4 carbon peak indicates the persistence of mannan. At 120 min, this signal disappears, confirming a more amorphous mannan structure, consistent with the XRD and FTIR data and suggesting enhanced enzymatic accessibility.

At 180 min, two small resolved signals in the anomeric carbon region became visible. At 240 min, these signals intensified along with the appearance of a signal near the C4 carbon (Figure 8A). This behavior over long mechanochemical times can indicate the hypothesis of a greater contribution of the second constituent in the biomass or minimal degradation of mannan, generating two signals of anomeric mannose carbons.

The Δν1/2 values measured for the six signals of the mannose skeleton increase with the grinding times, mainly for carbons C1 and C6, reflecting higher amorphization of the mannan fraction. The greater impact was observed for C1, the anomeric carbon, which increased from 171 to 492 Hz after 60 min of pretreatment. Other signals also showed polymorphic changes, although less pronounced than observed for C1. The characterization of the enzymatic residues of the pretreated samples is in progress and will be reported in future communication.

## Conclusions

The characterization of macauba seed cake confirmed it to be a rich source of d-mannose (44%) and a promising raw material for the carbohydrate market after oil extraction. The cake from ripe seeds predominantly contained linear mannan, typical of palm seeds, along with other biomolecules such as proteins and lignin (16.1%).

Ball milling effectively reduced mannan crystallinity (26.6% without pretreatment to 1.2% after 120 min and 2.4% after 180 min of ball milling pretreatment), induced polymorphic transformations, and enhanced polysaccharide bioavailability, facilitating enzymatic hydrolysis by mannanases. This led to high mannose release during the enzymatic hydrolysis (achieving 76.8% and 78.4% of mannose yield after 120 and 180 min of pretreatment, respectively), although modifications of the mechanochemical process may occur, with minimal mannan degradation and the contribution of the second constituent as well. These hypotheses may occur after 180 min of ball milling. Additionally, *A. niger* 1234 exhibited invertase activity, imparting trace amounts of glucose and fructose (1–5%) to the enzymatic hydrolysate.

Solid-state ^13^C CPMAS NMR proved to be highly sensitive for monitoring mannan I polymorphs, revealing amorphization and improved enzymatic accessibility. These results were consistent with complementary analytical techniques and correlated with mannose yield measured by DNS and HPLC. To our knowledge, this is the first study to report mannose production from macauba seed cake via ball milling and enzymatic hydrolysis and the first to demonstrate solid-state NMR as a tool to track amorphization in mannan-rich biomasses.

## Supplementary Material



## Notes

Macauba seed cake samples ground at different ball milling times; HPLC chromatograms of samples BM0-AH and reference mixture analyzed with a HPX-87P column; ^1^H NMR spectra of samples BM0-AH and d-mannose, d-glucose, d-galactose, d-xylose, and d-arabinose standards; ^13^C NMR spectra of samples BM0-AH, d-mannose and d-glucose standards; ^13^C and ^1^H NMR spectra of samples BM0-E and sucrose, d-glucose, and d-fructose standards; HPLC chromatograms of samples analyzed with a HPX-87P column; comparison between mannose concentration in the samples BM0, BM30, BM60, BM120, BM180, and BM240, analyzed with a HPX-87P column, and reducing the sugar concentration (DNS method); comparison between mannose concentration in the samples BM0, BM30, BM60, BM120, BM180, and BM240, analyzed with a HPX-87P column, and mannose yield (%); NMR spectra of sample BM120-control, sucrose, and d-cellobiose; ^1^H NMR spectra of the comparison between BM0 and the enzyme sample; NMR spectra of samples BM120, BM120-control, and BM0-E; NMR spectra of sample BM120 and d-mannose, d-glucose, and d-fructose standards; NMR spectra of d-mannose; NMR spectra of d-glucose; NMR spectra of d-fructose; NMR spectra of sucrose; ^13^C NMR data for samples BM0-AH and BM0-E; ^1^H NMR data of sample BM0-E; statistical analysis (Cochran’s test) of the calibration curve for reducing sugar quantification by the DNS method; ^1^H NMR data of samples BM120, BM120-control, and standards; ^13^C NMR data for samples BM120, BM120-control, and standards; representative absorptions observed by FTIR for the BM0 sample compared to standards and literature; representative absorptions observed by FTIR of the pretreated samples; and comparison of diffraction peaks observed for mannan I and cellulose I (PDF)
