# Supplementary material for: Influence of Mechanochemical Pretreatment on the Bioavailability of Mannan from Macauba Seed Cake
Source: ACS Omega. 2026 Jun 13;11(25):36694–710. doi: 10.1021/acsomega.5c12892 (PMC13325103; doi:10.1021/acsomega.5c12892)
Supplement: Supplementary file 1 [file ao5c12892_si_001.pdf]

Supplementary Material

Influence of Mechanochemical Pretreatment on the Bioavailability of Mannan from Macauba Seed Cake

Michelle Ramos Cavalcante Fortunato[a,b] , Rosane Aguiar da Silva San Gil[a] , Leandro Bandeira Borre[a] , Ricardo Sposina Sobral Teixeira[b,*]

[a] *Universidade Federal do Rio de Janeiro, Institute of Chemistry, Solid State NMR Lab., RJ, Brazil.*
[b] *Universidade Federal do Rio de Janeiro, Institute of Chemistry, Bioethanol Lab., Rio de Janeiro, Brazil.*
**author for correspondence : ricardot@iq.ufrj.br*

**Figure S1**	Macauba endosperm cake samples ground at different ball milling times.
**Figure S2**	HPLC chromatograms of samples BM0-AH and reference mixture analyzed with HPX-87P column.
**Figure S3**	^{1}H NMR spectra (500MHz, D_2O+DSS) of samples BM0-AH and D-mannose, D-glucose, D-galactose, D-xylose, D-arabinose standards with water signal suppression, in the region 5.6-3.0 ppm (bottom); expanded anomeric H region 5.6-4.4 ppm (top).
**Figure S4**	^{13}C NMR spectra (125 MHz, D_2O+DSS) of samples BM0-AH, D-mannose and D-glucose standards.
**Figure S5**	^{13}C NMR spectra (125 MHz, D_2O+DSS) of samples BM0-E and sucrose, D-glucose and D-fructose standards (top); ^{1}H NMR spectra (500MHz, D_2O+DSS) of samples BM0-E and sucrose, D-glucose and D-fructose standards with water signal suppression (bottom).
**Figure S6**	HPLC chromatograms of samples analyzed with HPX-87P column. (A) control samples; (B) samples after 72h of enzymatic hydrolysis.
**Figure S7**	Comparison between mannose concentration in the samples BM0, BM30, BM60, BM120, BM180 and BM240, analyzed with HPX-87P column, and reducing sugar concentration (DNS method).
**Figure S8**	Comparison between mannose concentration in the samples BM0, BM30, BM60, BM120, BM180 and BM240, analyzed with HPX-87P column, and mannose yield (%).
**Figure S9**	NMR spectra of sample BM120-control: (bottom) ^{1}H spectra (500 MHz, D_2O+DSS) of BM120-control, sucrose and D-cellobiose with water signal suppression; (top) ^{13}C spectra (125 MHz, D_2O+DSS) of BM120-control, sucrose and D-cellobiose standards.

**Figure S10**	^{1}H NMR spectra (500 MHz, D_2O+DSS) of the comparison between BM0 and enzyme sample, with water signal suppression.
**Figure S11**	NMR spectra of samples BM120, BM120-control and BM0-E: (bottom) ^{1}H spectra (500MHz, D_2O+DSS) with water signal suppression; (top) ^{13}C NMR spectra (125MHz, D_2O+DSS).
**Figure S12**	NMR spectra of sample BM120: (bottom) ^{1}H spectra (500MHz, D_2O+DSS) of BM120 and D-mannose, D-glucose, D-fructose standards, with water signal suppression; (top) ^{13}C spectrum (125MHz, D_2O+DSS), of BM120 and D-mannose, D-glucose, D-fructose standards.
**Figure S13**	NMR spectra of D-mannose: (bottom) ^{1}H spectrum (500MHz, D_2O+DSS) with water signal suppression; (top) ^{13}C spectrum (125MHz, D_2O+DSS).
**Figure S14**	NMR spectra of D-glucose: (bottom) ^{1}H spectrum (500MHz, D_2O+DSS) with water signal suppression; (top) ^{13}C spectrum (125MHz, D_2O+DSS).
**Figure S15**	NMR spectra of D-fructose: (bottom) ^{1}H spectrum (500MHz, D_2O+DSS) with water signal suppression; (top) ^{13}C spectrum (125MHz, D_2O+DSS).
**Figure S16**	NMR spectra of sucrose: (bottom) ^{1}H spectrum (500MHz, D_2O+DSS) with water signal suppression; (top) ^{13}C spectrum (125MHz, D_2O+DSS).
**Table S1**	^{13}C NMR data (125MHz, D_2O+DSS) for samples BM0-AH and BM0-E.
**Table S2**	^{1}H NMR data (500MHz, D_2O+DSS) of sample BM0-E.
**Table S3**	Statistical analysis (Cochran's test) of calibration curve for reducing sugar quantification by DNS method.
**Table S4**	^{1}H NMR data (500MHz, D_2O+DSS) of samples BM120, BM120-control, and standards.
**Table S5**	^{13}C NMR data (125MHz, D_2O+DSS) for samples BM120, BM120-control and standards.
**Table S6**	Representative absorptions observed by FTIR for the BM0 sample compared to standards and literature.
**Table S7**	Representative absorptions observed by FTIR of the pretreated samples.
**Table S8**	Comparison of diffraction peaks observed for mannan I and cellulose I.
**References**	Table references.

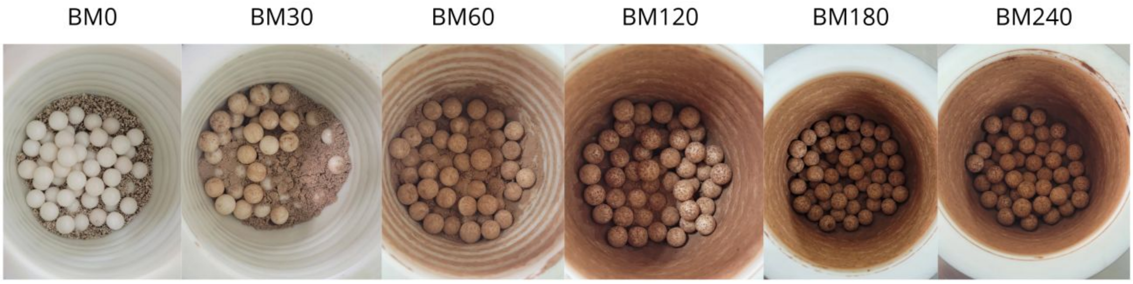

Figure S1. Macauba endosperm cake samples ground at different ball milling times.

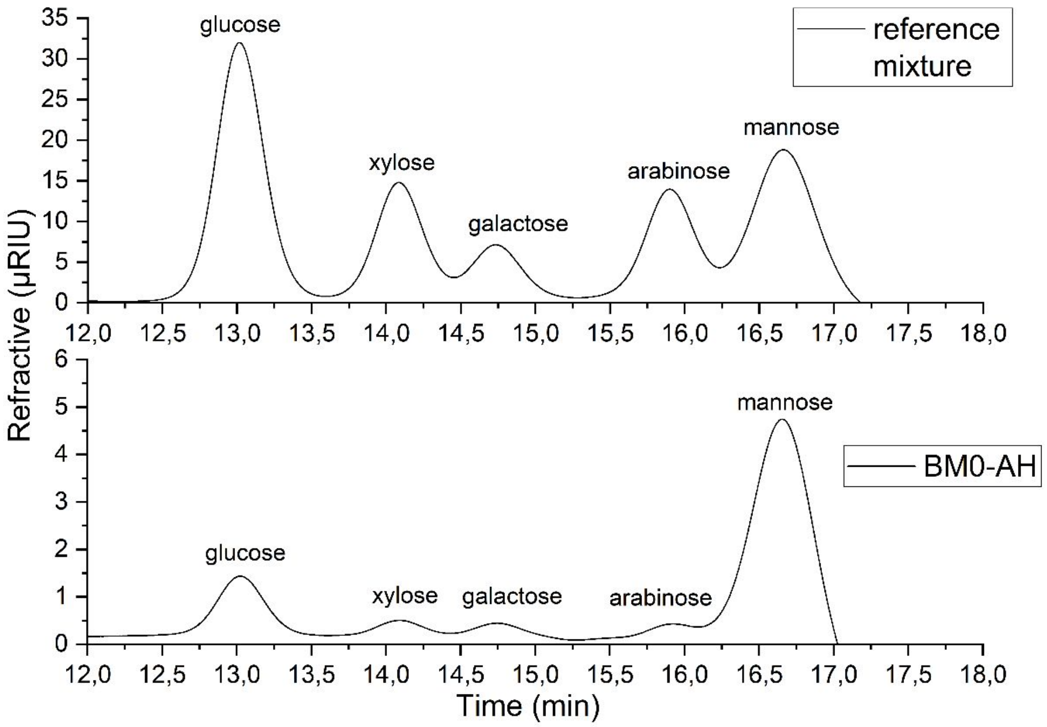

Figure S2. HPLC chromatograms of samples BM0-AH and reference mixture analyzed with HPX-87P column.

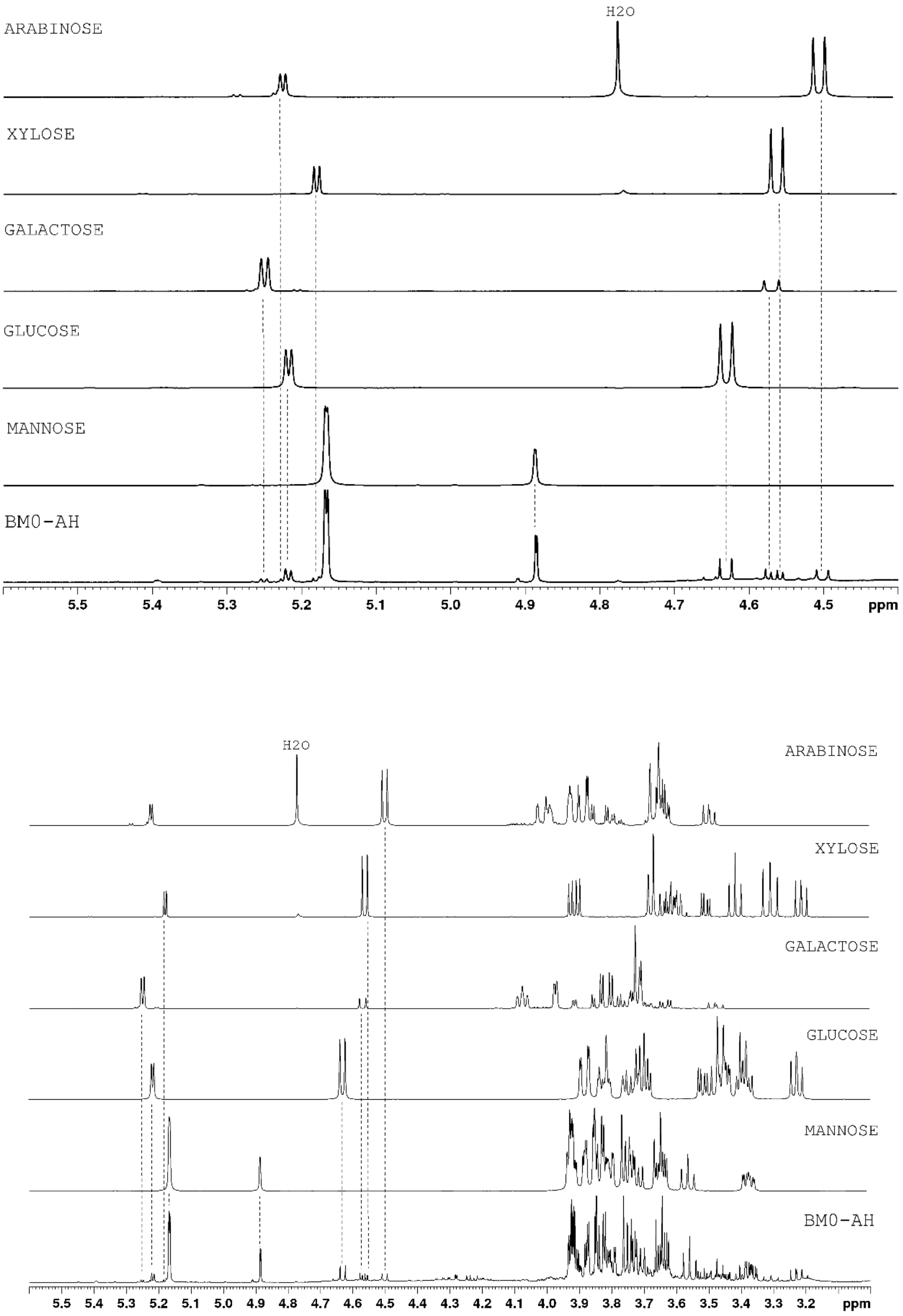

Figure S3. ^{1}H NMR spectra (500MHz, D_2O+DSS) of samples BM0-AH and D-mannose, D-glucose, D-galactose, D-xylose, D-arabinose standards with water signal suppression in the region 5.6-3.0 ppm (bottom); expanded anomeric H region 5.6-4.4 ppm (top).

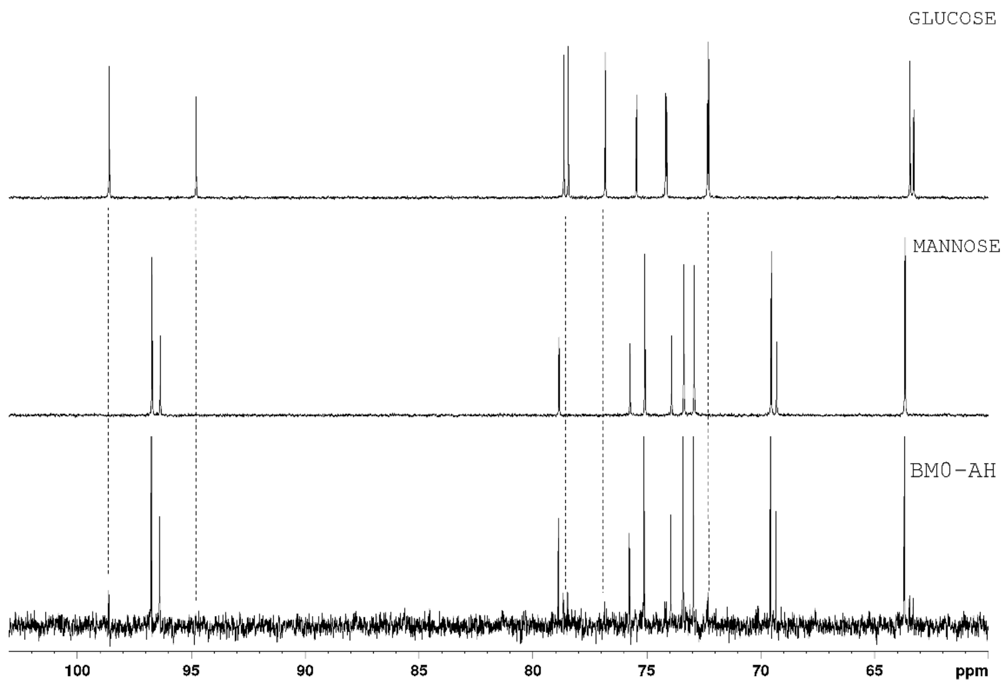

Figure S4. ^{13}C NMR spectra (125 MHz, D_2O+DSS) of samples BM0-AH, D-mannose and D-glucose standards.

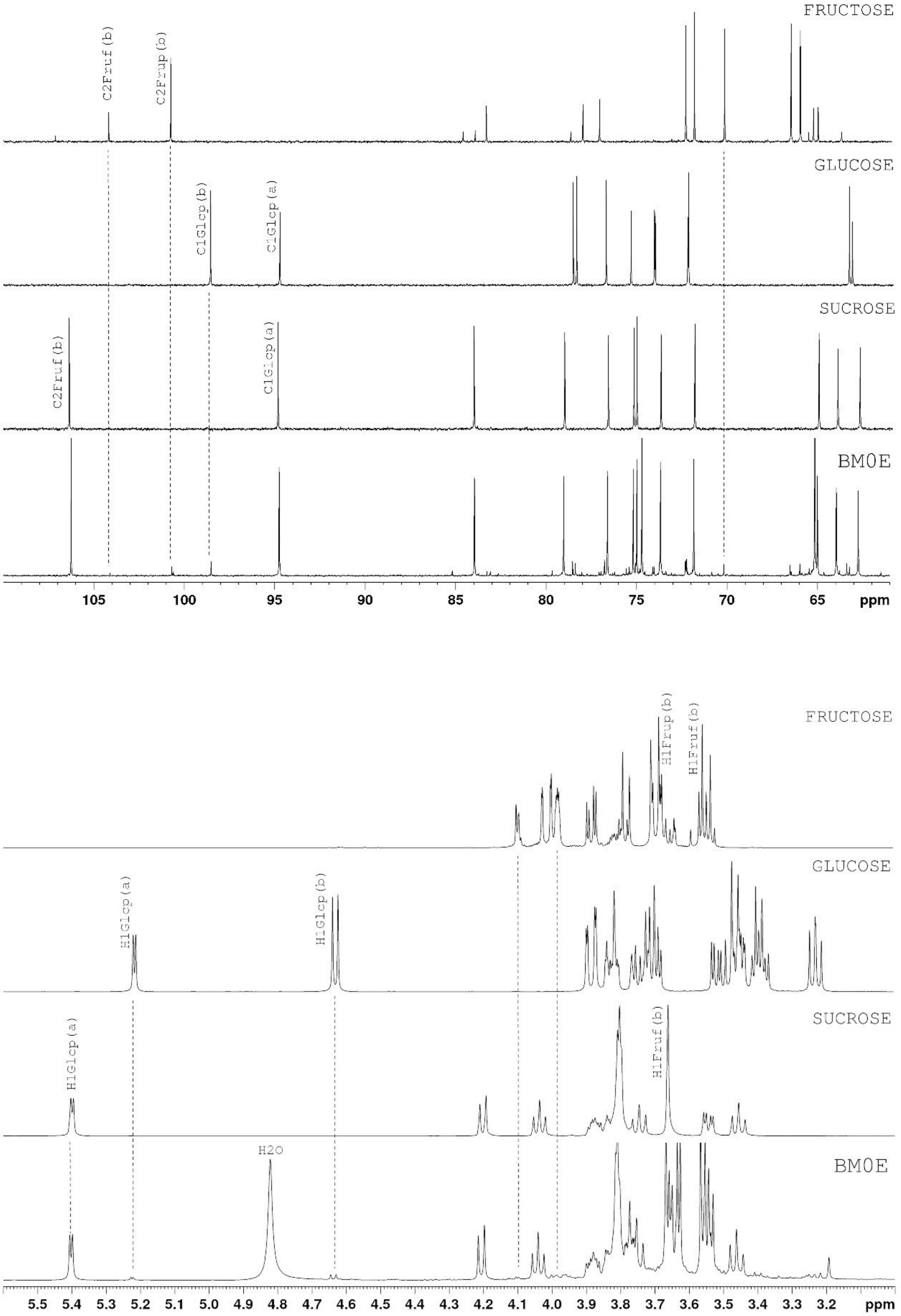

Figure S5. ^{13}C NMR spectra (125 MHz, D_2O+DSS) of samples BM0-E and sucrose, D-glucose and D-fructose standards (top); ^{1}H NMR spectra (500MHz, D_2O+DSS) of samples BM0-E and sucrose, D-glucose and D-fructose standards with water signal suppression (bottom).

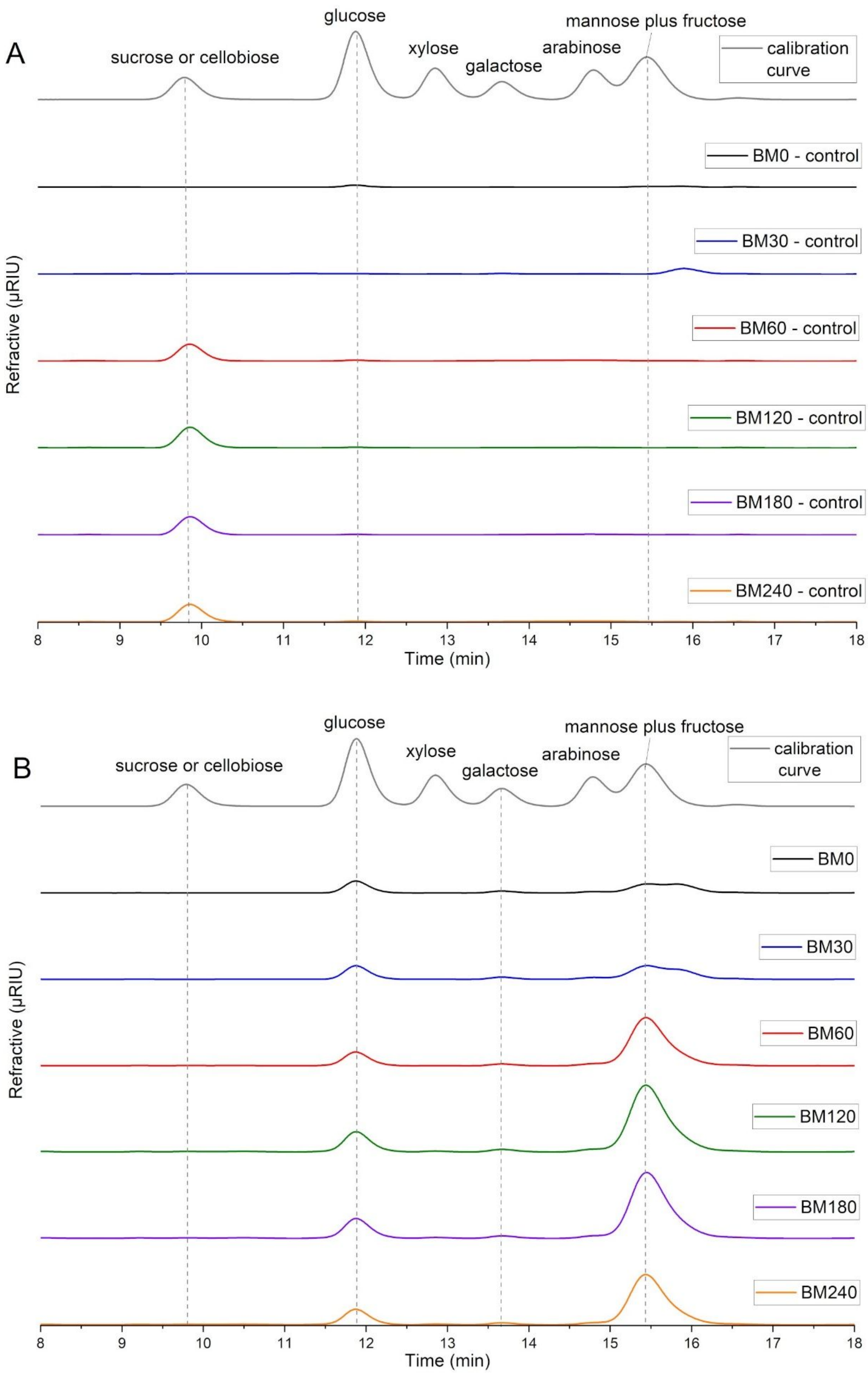

Figure S6. HPLC chromatograms of samples analyzed with HPX-87P column. (A) control samples; (B) samples after 72h of enzymatic hydrolysis.

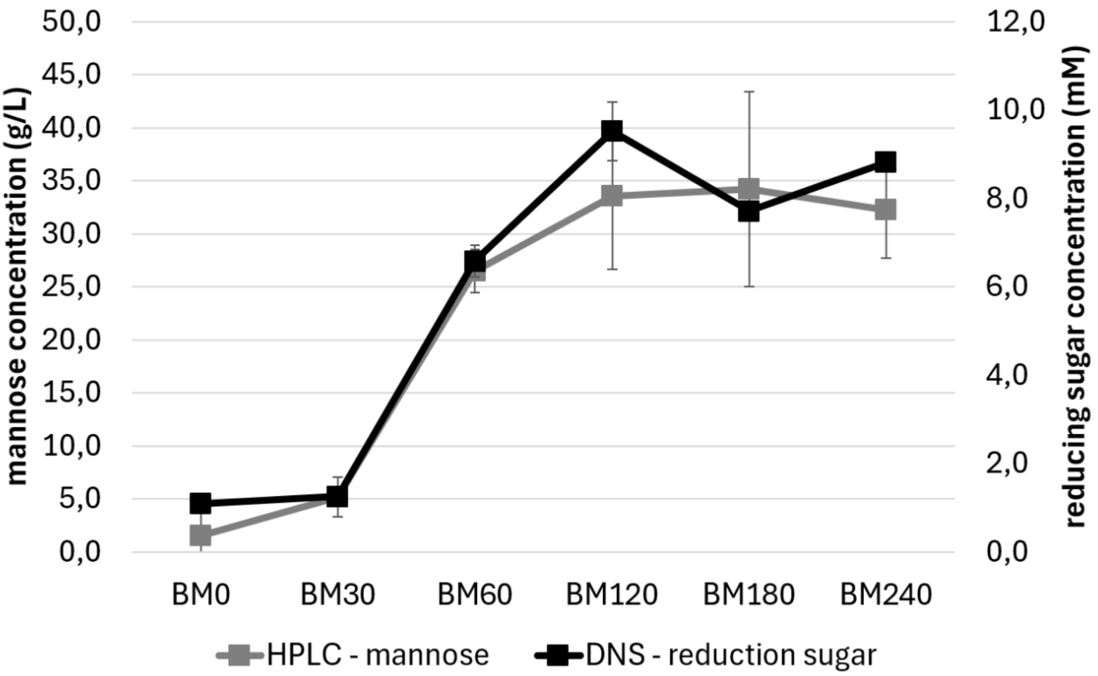

Figure S7. Comparison between mannose concentration in the samples BM0, BM30, BM60, BM120, BM180 and BM240, analyzed with HPX-87P column, and reducing sugar concentration (DNS method).

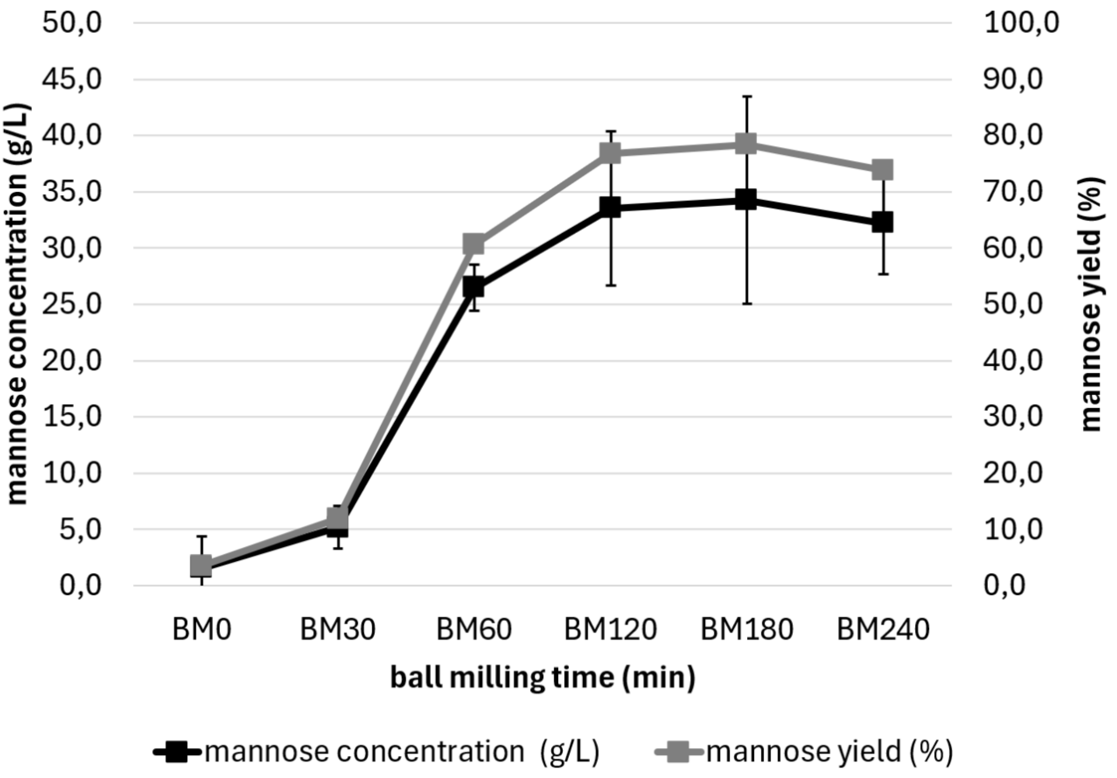

Figure S8. Comparison between mannose concentration in the samples BM0, BM30, BM60, BM120, BM180 and BM240, analyzed with HPX-87P column, and mannose yield (%).

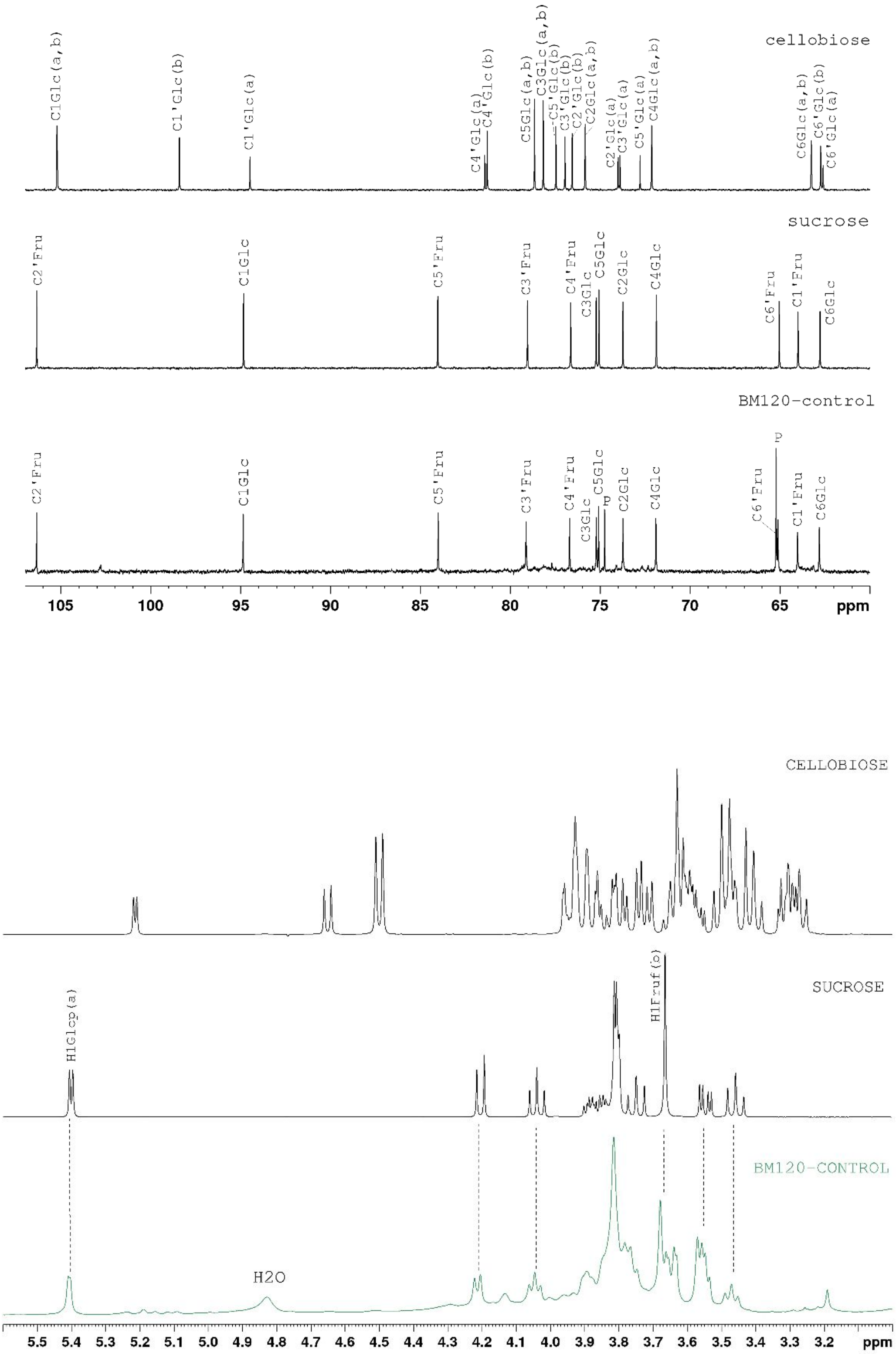

Figure S9. NMR spectra of sample BM120-control: (bottom) ^{1}H spectra (500 MHz, D_2O+DSS) of BM120-control, sucrose and D-cellobiose with water signal suppression; (top) ^{13}C spectra (125 MHz, D_2O+DSS) of BM120-control, sucrose and D-cellobiose standards.

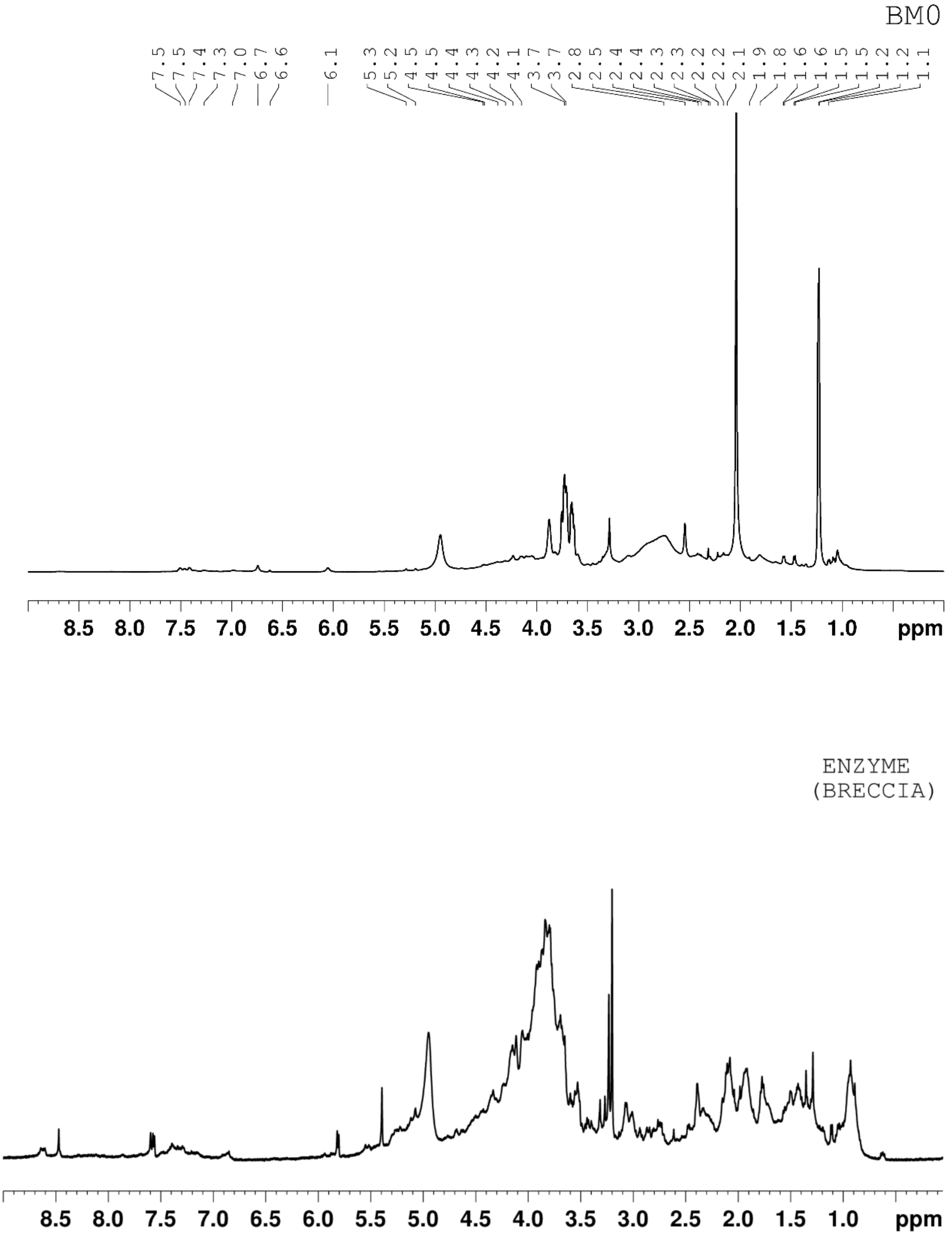

Figure S10. ^{1}H NMR spectra (500 MHz, D_2O+DSS) of the comparison between BM0 and enzyme sample, with water signal suppression.

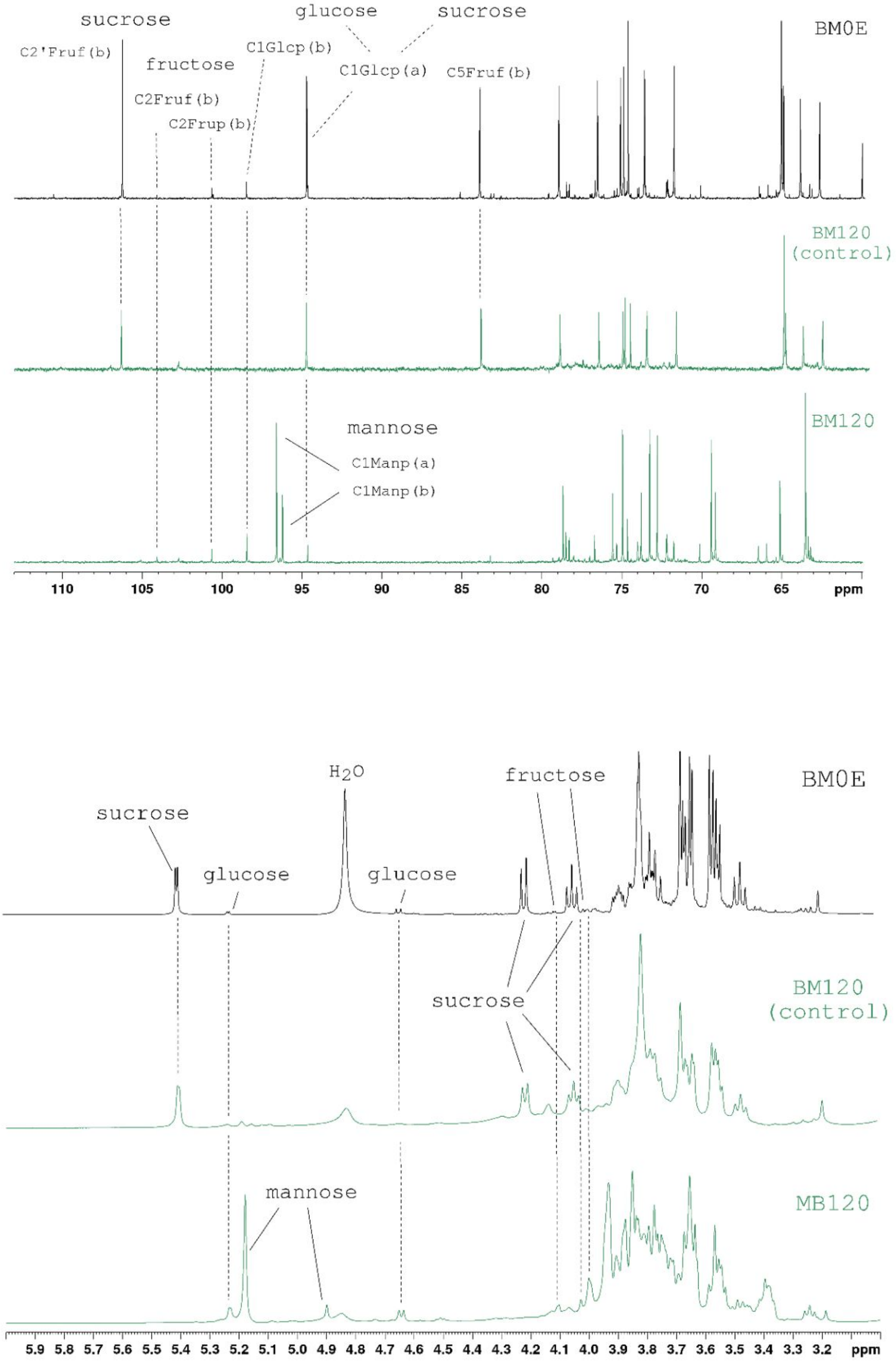

Figure S11. NMR spectra of samples BM120, BM120-control and BM0-E: (bottom) ^{1}H spectra (500MHz, D_2O+DSS) with water signal suppression; (top) ^{13}C NMR spectra (125 MHz, D_2O+DSS).

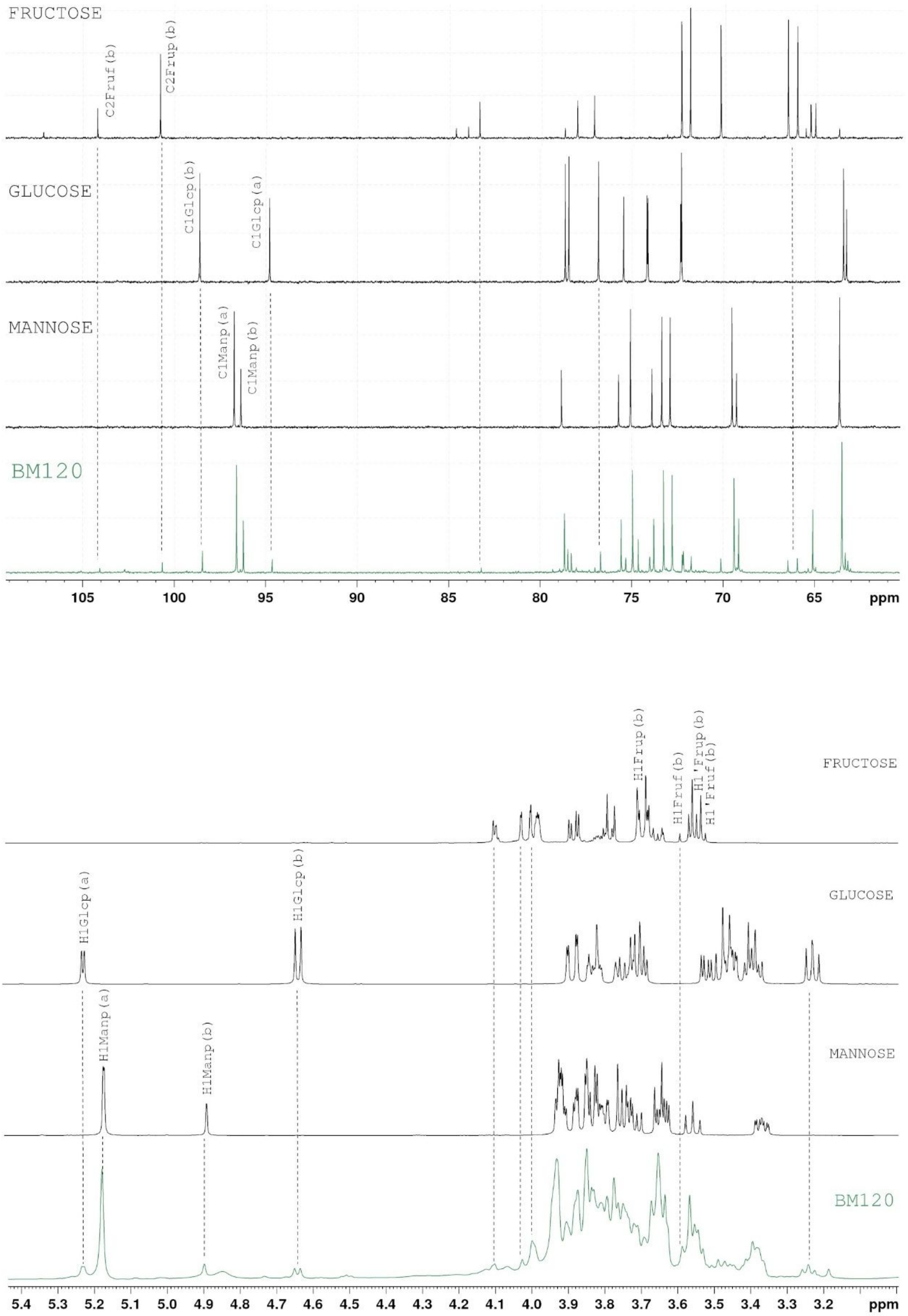

Figure S12. NMR spectra of sample BM120: (bottom) ^{1}H spectra (500MHz, D_2O+DSS) of BM120 and D-mannose, D-glucose, D-fructose standards, with water signal suppression; (top) ^{13}C spectrum (125MHz, D_2O+DSS) of BM120 and D-mannose, D-glucose, D-fructose standards.

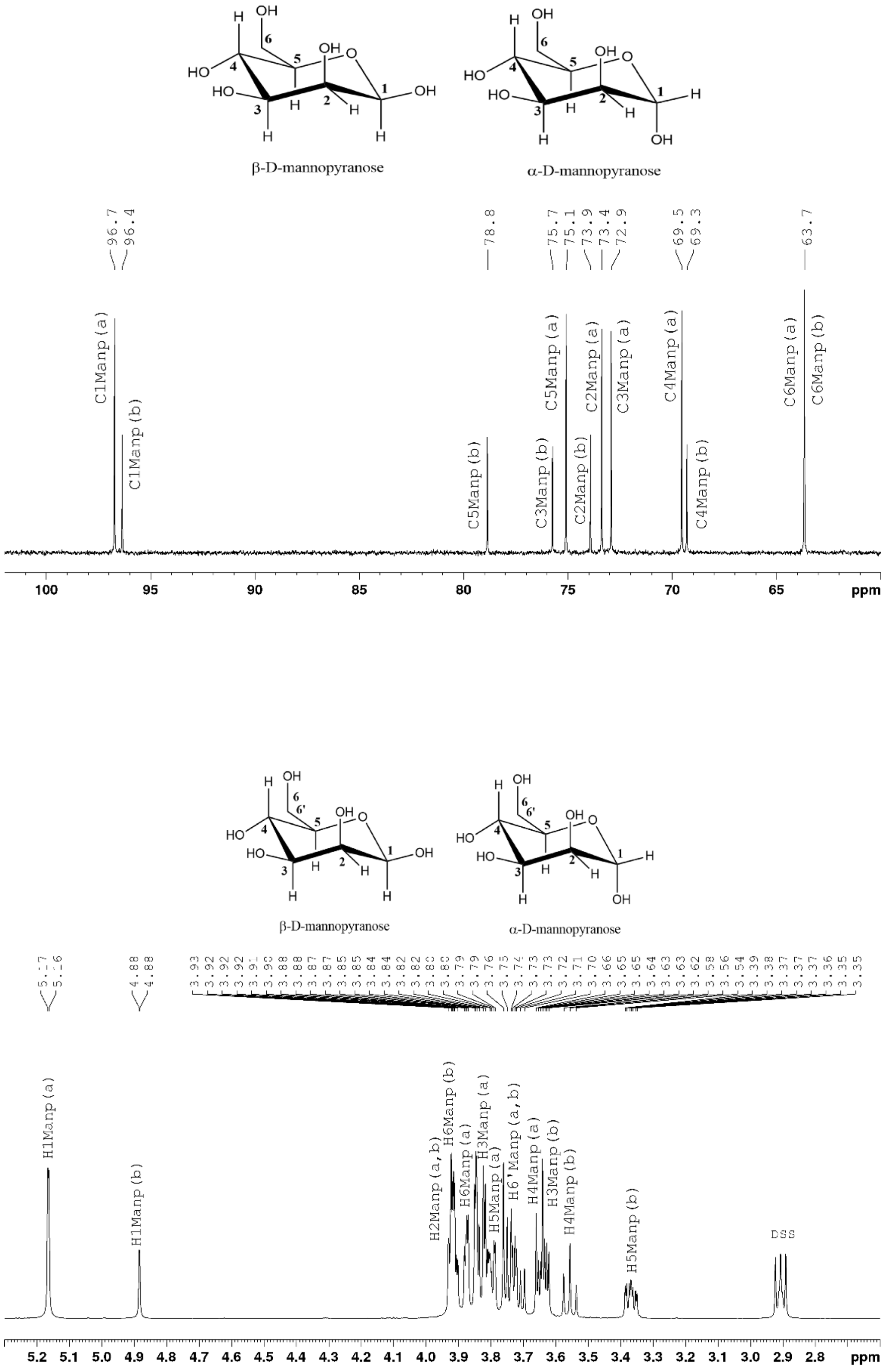

Figure S13. NMR spectra of D-mannose: (bottom) ^{1}H spectrum (500MHz, D_2O+DSS) with water signal suppression; (top) ^{13}C spectrum (125MHz, D_2O+DSS).

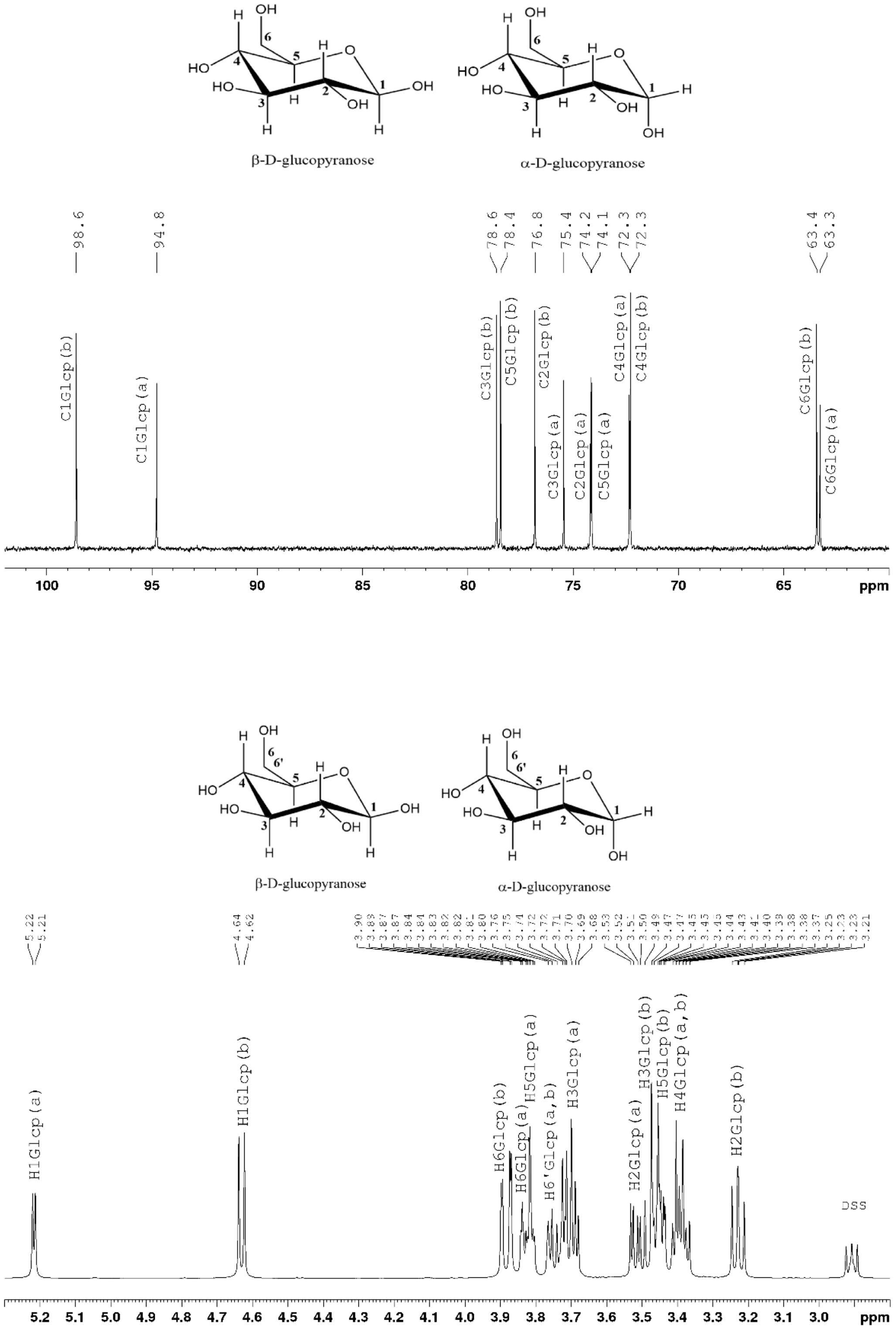

Figure S14. NMR spectra of D-glucose: (bottom) ^{1}H spectrum (500MHz, D_2O+DSS) with water signal suppression; (top) ^{13}C spectrum (125MHz, D_2O+DSS).

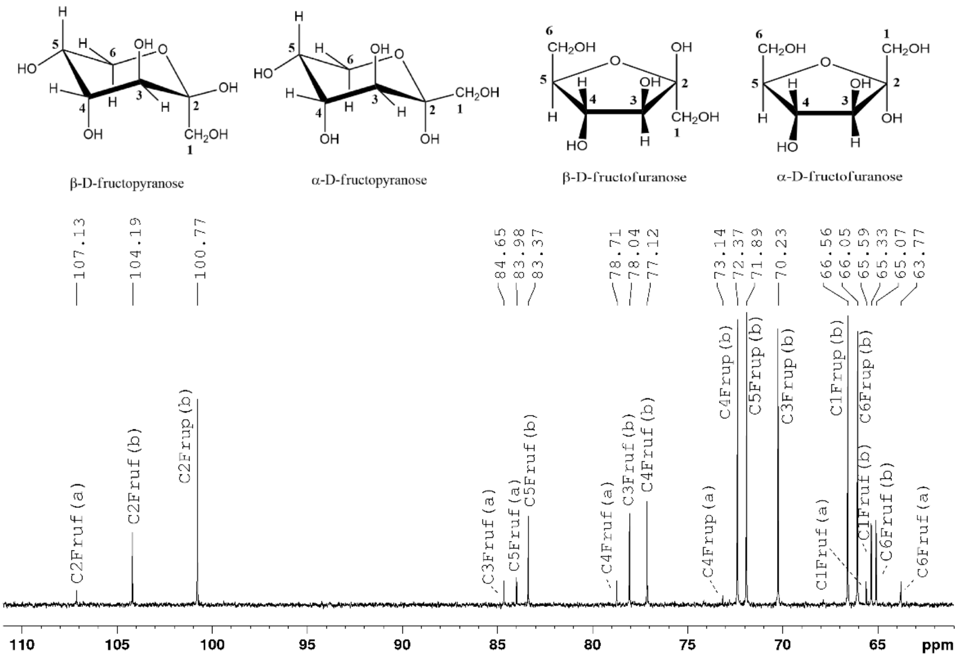

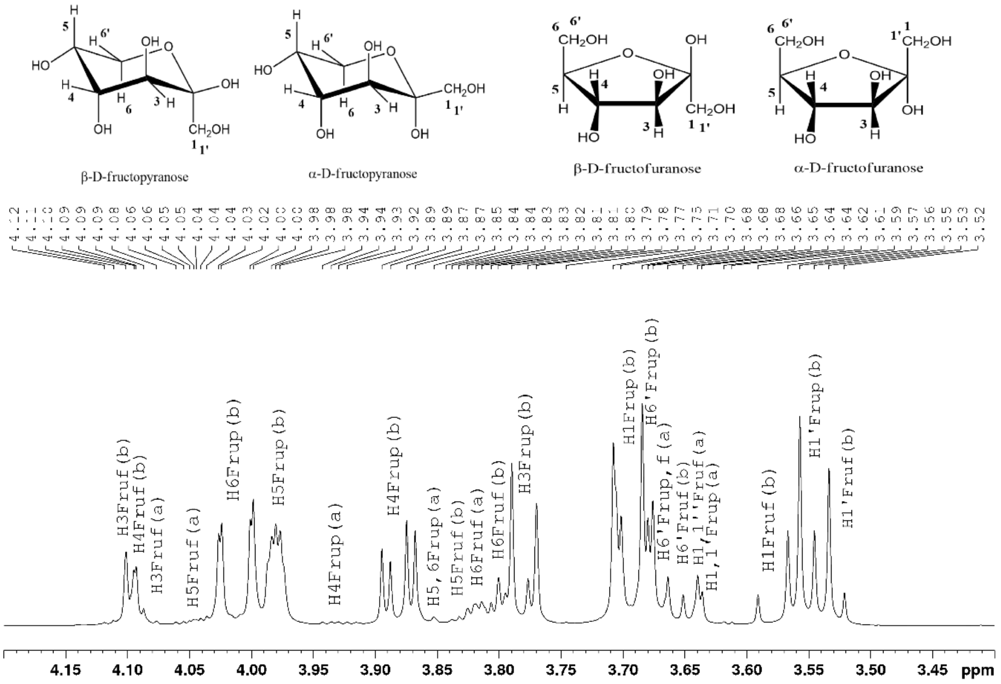

Figure S15. NMR spectra of D-fructose: (bottom) ^{1}H spectrum (500MHz, D_2O+DSS) with water signal suppression; (top) ^{13}C spectrum (125MHz, D_2O+DSS).

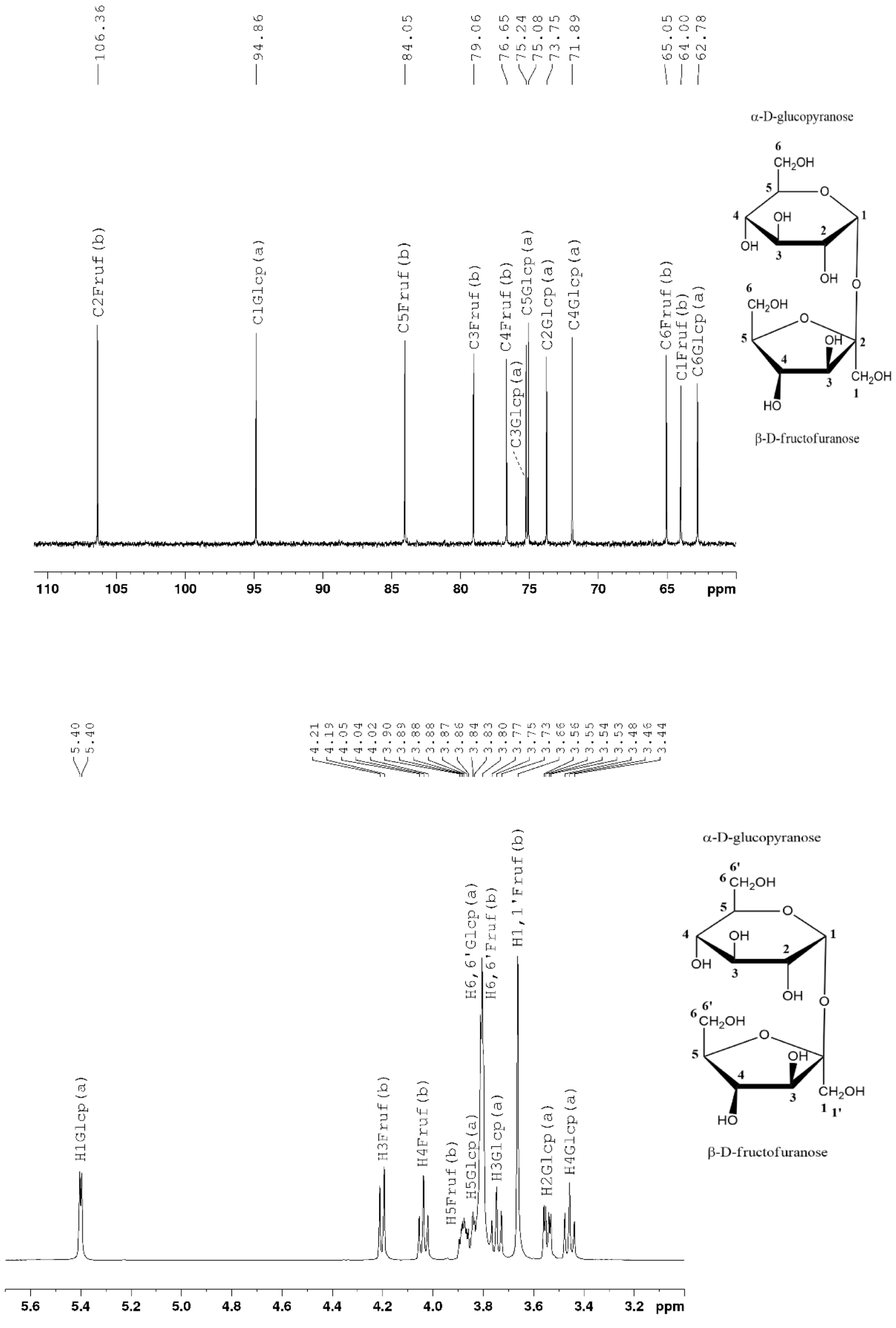

Figure S16. NMR spectra of sucrose: (bottom) ^{1}H spectrum (500MHz, D_2O+DSS) with water signal suppression; (top) ^{13}C spectrum (125MHz, D_2O+DSS).

Table S1. ^{13}C NMR data (125MHz, D_2O+DSS) for samples BM0-AH and BM0-E.

samples	composition		δ(ppm)						Reference
			**C1**	**C2**	**C3**	**C4**	**C5**	**C6**	
BM0E	sucrose	α-D-glucopyranose	94.8	73.7	75.2	71.8	75.0	62.7	this work
			94.9<br>(92.9)	73.7<br>(72.0)	75.2<br>(73.6)	71.9<br>(70.2)	75.1<br>(73.3)	62.8<br>(61.1)	standard - this work<br>(1)
		β-D-fructofuranose	64.0	106.3	79.0	76.6	84.0	65.1	this work
			64.0<br>(63.3)	106.4<br>(104.4)	79.1<br>(77.4)	76.7<br>(75.0)	84.0<br>(82.2)	65.0<br>(63.4)	standard - this work<br>(1)
	D-glucose	α-D-glucopyranose	94.8	74.1	75.4	72.3	74.1	63.2	this work
			94.8<br>(93.6)	74.2<br>(73.2)	75.4<br>(74.5)	72.3<br>(71.4)	74.1<br>(73.0)	63.3<br>(62.3)	standard - this work<br>(1)
		β-D-glucopyranose	98.5	76.8	78.5	72.3	78.4	63.4	this work
			98.6<br>(97.4)	76.8<br>(75.9)	78.6<br>(77.5)	72.3<br>(71.3)	78.4<br>(77.4)	63.4<br>(62.5)	standard - this work<br>(1)
	D-fructose	β-D-fructopyranose	66.5	100.7	70.2	72.3	71.9	66.0	this work
			66.6<br>(64.7)	100.8<br>(99.1)	70.2<br>(68.4)	72.4<br>(70.5)	71.9<br>(70.0)	66.0<br>(64.1)	standard - this work<br>(1)
		β-D-fructofuranose	65.3	104.1	78.0	77.1	83.3	63.8	this work
			63.3<br>(63.6)	104.2<br>(102.6)	78.0<br>(76.4)	77.1<br>(75.4)	83.4<br>(81.6)	65.1<br>(63.2)	standard - this work<br>(1)
BM0-AH	D-mannose	α-D-mannopyranose	96.7	73.4	73.0	69.5	75.1	63.7	this work
			96.7<br>(95.5)	73.4<br>(72.3)	72.9<br>(71.9)	69.5<br>(68.5)	75.1<br>(73.9)	63.7<br>(62.6)	standard - this work<br>(1)
		β-D-mannopyranose	96.4	73.9	75.8	69.3	78.9	63.7	this work
			96.4<br>(95.2)	73.9<br>(72.8)	75.7<br>(74.8)	69.3<br>(68.3)	78.8<br>(77.6)	63.7<br>(62.6)	standard - this work<br>(1)
	D-glucose	α-D-glucopyranose	*	74.2	*	72.3	74.1	63.3	this work
			94.8<br>(93.6)	74.2<br>(73.2)	75.4<br>(74.5)	72.3<br>(71.4)	74.1<br>(73.0)	63.3<br>(62.3)	standard - this work<br>(1)
		β-D-glucopyranose	98.6	76.8	78.6	72.3	78.4	63.4	this work
			98.6<br>(97.4)	76.8<br>(75.9)	78.6<br>(77.5)	72.3<br>(71.3)	78.4<br>(77.4)	63.4<br>(62.5)	standard - this work<br>(1)

*not detected

Table S2. ^{1}H NMR data (500 MHz, D_2O+DSS) of sample BM0-E.

samples	composition		δ(ppm)								Reference
			**H1**	**H1'**	**H2**	**H3**	**H4**	**H5**	**H6**	**H6'**	
BM0-E	sucrose	α-D-glucopyranose	5.402	-	3.548	3.753	3.462	3.845	3.809	3.809	this work
			5.400<br>(5.38)	-	3.544<br>(3.52)	3.747<br>(3.72)	3.457<br>(3.43)	3.843<br>(3.83)	3.805<br>(3.78)	3.805<br>(3.78)	standard - this work<br>(2)
		β-D-fructofuranose	3.668	3.668	-	4.206	4.040	3.884	3.809	3.809	this work
			3.662<br>(3.63)	3.662<br>(3.63)	-	4.203<br>(4.18)	4.038<br>(4.01)	3.881<br>(3.86)	3.805<br>(3.79)	3.805<br>(3.79)	standard - this work<br>(2)
	D-glucose	α-D-glucopyranose	5.224	-	3.523	3.698	3.399	*	3.843	*	this work
			5.217<br>(5.214)<br>(5.21)	-	3.518<br>(3.516)<br>(3.52)	3.700<br>(3.696)<br>(3.70)	3.394<br>(3.393)<br>(3.39)	3.816<br>(3.817)<br>(3.81)	3.838<br>(3.823)<br>(3.84)	3.745<br>(3.745)<br>(3.75)	standard - this work<br>(3)<br>(4)
		β-D-glucopyranose	4.639	-	3.234	*	3.399	*	3.890	*	this work
			4.630<br>(4.627)<br>(4.63)	-	3.228<br>(3.226)<br>(3.23)	3.473<br>(3.469)<br>(3.46)	3.385<br>(3.385)<br>(3.38)	3.450<br>(3.447)<br>(3.44)	3.883<br>(3.879)<br>(3.89)	3.702<br>(3.704)<br>(3.70)	standard - this work<br>(3)<br>(4)
	D-fructose	β-D-fructopyranose	*	*	-	*	*	3.987	4.010	*	this work
			3.696<br>(3.71)	3.546<br>(3.56)	-	3.780<br>(3.80)	3.882<br>(3.90)	3.981<br>(4.00)	4.013<br>(4.03)	3.689<br>(3.71)	standard - this work<br>(5)
		β-D-fructofuranose	*	*	-	4.101	*	*	*	*	this work
			3.579<br>(3.59)	3.534<br>(3.55)	-	4.098<br>(4.12)	4.099<br>(4.12)	3.820<br>(3.85)	3.789<br>(3.81)	3.652<br>(3.68)	standard - this work<br>(5)
			5.165<br>(5.05)	-	3.917<br>(3.80)	3.830<br>(3.71)	3.641<br>(3.52)	3.799<br>(3.68)	3.860<br>(3.74)	3.744<br>(3.62)	standard - this work<br>(4)
			4.884<br>(4.77)	-	3.927<br>(3.82)	3.641<br>(3.53)	3.556<br>(3.45)	3.368<br>(3.26)	3.892<br>(3.78)	3.715<br>(3.60)	standard - this work<br>(4)
			5.217<br>(5.214)<br>(5.21)	-	3.518<br>(3.516)<br>(3.52)	3.700<br>(3.696)<br>(3.70)	3.394<br>(3.393)<br>(3.39)	3.816<br>(3.817)<br>(3.81)	3.838<br>(3.823)<br>(3.84)	3.745<br>(3.745)<br>(3.75)	standard - this work<br>(3)<br>(4)
			4.630<br>(4.627)<br>(4.63)	-	3.228<br>(3.226)<br>(3.23)	3.473<br>(3.469)<br>(3.46)	3.385<br>(3.385)<br>(3.38)	3.450<br>(3.447)<br>(3.44)	3.883<br>(3.879)<br>(3.89)	3.702<br>(3.704)<br>(3.70)	standard - this work<br>(3)<br>(4)
			(5.26)	-	(3.8)	(3.83)	(4.12)	(3.79)	(5.09)	(5.09)	standard - this work<br>(6)
			(4.57)	-	(3.49)	(3.62)	(4.06)	(3.40)	(5.12)	(5.12)	standard - this work<br>(6)

*not resolved

Table S3. Statistical analysis (Cochran's test) of calibration curve for mannose quantification by DNS method.

Cochran's test						
replicate (*n*)	df	significance level (alpha)	maximum variance	sum variance	*C*	*C crit*
3	5	0.05	0.001567	0.002848	0.550	0.684
C < *C crit* (homogeneous variances - homoscedastic)						

Table S4. ^{1}H NMR data (500 MHz, D_2O+DSS) of samples BM120, BM120-control, and standards.

samples	composition		δ(ppm) H1	H1'	H2	H3	H4	H5	H6	H6'	Reference
BM120 – control	sucrose	α-D-glucopyranose	5.406	-	3.552	3.765	3.470	3.893	3.830	3.830	this work
			5.400<br>(5.38)	-	3.544<br>(3.52)	3.747<br>(3.72)	3.457<br>(3.43)	3.843<br>(3.83)	3.805<br>(3.78)	3.805<br>(3.78)	standard - this work<br>(2)
		β-D-fructofuranose	3.678	3.678	-	4.213	4.046	3.968	3.830	3.830	this work
			3.662<br>(3.63)	3.662<br>(3.63)	-	4.203<br>(4.18)	4.038<br>(4.01)	3.881<br>(3.86)	3.805<br>(3.79)	3.805<br>(3.79)	standard - this work<br>(2)
BM120	D-mannose	α-D-mannopyranose	5.179	-	*	3.842	3.654	3.802	3.864	3.763	this work
			5.165<br>(5.05)	-	3.917<br>(3.80)	3.830<br>(3.71)	3.641<br>(3.52)	3.799<br>(3.68)	3.860<br>(3.74)	3.744<br>(3.62)	standard - this work<br>(4)
		β-D-mannopyranose	4.898	-	3.941	3.654	3.567	3.380	3.932	*	this work
			4.884<br>(4.77)	-	3.927<br>(3.82)	3.641<br>(3.53)	3.556<br>(3.45)	3.368<br>(3.26)	3.892<br>(3.78)	3.715<br>(3.60)	standard - this work<br>(4)
	D-glucose	α-D-glucopyranose	5.231	-	*	3.692	3.396	*	*	*	this work
			5.217<br>(5.214)<br>(5.21)	-	3.518<br>(3.516)<br>(3.52)	3.700<br>(3.696)<br>(3.70)	3.394<br>(3.393)<br>(3.39)	3.816<br>(3.817)<br>(3.81)	3.838<br>(3.823)<br>(3.84)	3.745<br>(3.745)<br>(3.75)	standard - this work<br>(3)<br>(4)
		β-D-glucopyranose	4.643	-	3.243	3.489	3.396	3.474	3.906	3.906	this work
			4.630<br>(4.627)<br>(4.63)	-	3.228<br>(3.226)<br>(3.23)	3.473<br>(3.469)<br>(3.46)	3.385<br>(3.385)<br>(3.38)	3.450<br>(3.447)<br>(3.44)	3.883<br>(3.879)<br>(3.89)	3.702<br>(3.704)<br>(3.70)	standard - this work<br>(3)<br>(4)
	D-fructose	β-D-fructopyranose	*	*	-	*	*	3.993	4.027	*	this work
			3.696<br>(3.71)	3.546<br>(3.56)	-	3.780<br>(3.80)	3.882<br>(3.90)	3.981<br>(4.00)	4.013<br>(4.03)	3.689<br>(3.71)	standard - this work<br>(5)
		β-D-fructofuranose	*	*	-	4.102	*	*	*	*	this work
			3.579<br>(3.59)	3.534<br>(3.55)	-	4.098<br>(4.12)	4.099<br>(4.12)	3.820<br>(3.85)	3.789<br>(3.81)	3.652<br>(3.68)	standard - this work<br>(5)

*not resolved.

Table S5. ^{13}C NMR data (125MHz, D_2O+DSS) for samples BM120, BM120-control and standards.

samples	composition		δ(ppm) C1	C2	C3	C4	C5	C6	Reference
BM120 – control	sucrose	α-D-glucopyranose	94.9	73.7	75.2	71.9	75.1	62.8	this work
			94.9 (92.9)	73.7 (72.0)	75.2 (73.6)	71.9 (70.2)	75.1 (73.3)	62.8 (61.1)	standard - this work (1)
		β-D-fructofuranose	64.0	106.3	79.1	76.7	84.0	65.1	this work
			64.0 (63.3)	106.4 (104.4)	79.1 (77.4)	76.7 (75.0)	84.0 (82.2)	65.0 (63.4)	standard - this work (1)
BM120	D-mannose	α-D-mannopyranose	96.6	73.2	72.8	69.4	74.9	63.5	this work
			96.7 (95.5)	73.4 (72.3)	72.9 (71.9)	69.5 (68.5)	75.1 (73.9)	63.7 (62.6)	standard - this work (1)
		β-D-mannopyranose	96.2	73.8	75.6	69.1	78.7	63.5	this work
			96.4 (95.2)	73.9 (72.8)	75.7 (74.8)	69.3 (68.3)	78.8 (77.6)	63.7 (62.6)	standard - this work (1)
	D-glucose	α-D-glucopyranose	94.6	74.0	75.3	72.2	74.0	63.2	this work
			94.8 (93.6)	74.2 (73.2)	75.4 (74.5)	72.3 (71.4)	74.1 (73.0)	63.3 (62.3)	standard - this work (1)
		β-D-glucopyranose	98.4	76.7	78.5	72.2	78.3	63.3	this work
			98.6 (97.4)	76.8 (75.9)	78.6 (77.5)	72.3 (71.3)	78.4 (77.4)	63.4 (62.5)	standard - this work (1)
	D-fructose	β-D-fructopyranose	66.4	100.6	70.1	72.2	71.7	65.9	this work
			66.6 (64.7)	100.8 (99.1)	70.2 (68.4)	72.4 (70.5)	71.9 (70.0)	66.0 (64.1)	standard - this work (1)
		β-D-fructofuranose	65.3	104.1	78.0	77.0	83.2	64.9	this work
			65.3 (63.6)	104.2 (102.6)	78.0 (76.4)	77.1 (75.4)	83.4 (81.6)	65.1 (63.2)	standard - this work (1)
		α-D-fructopyranose	*	*	*	*	*	*	this work
			* (65.9)	* (99.1)	* (70.9)	73.1 (71.3)	* (62.0)	* (61.9)	standard - this work (1)
		α-D-fructofuranose	*	*	*	*	*	*	this work
			65.6 (63.7)	107.1 (105.5)	84.6 (82.9)	78.7 (77.0)	84.0 (82.2)	63.8 (61.9)	standard - this work (1)

*not detected.

Table S6. Representative absorptions observed by FTIR for the BM0 sample compared to standards and literature.

region N^0	functional groups	assignments	wavenumber (cm^{-1})			
			BM0	mannan	cellulose	reference
**1**	alcohol	ν_{O-H} stretching O-H (intramolecular)	3385	3503<br>3475<br>3374	3408	3400-3300 [7]<br>3550-3200 [8]
**2**	amide	ν_{N-H} stretching N-H (asymmetric and symmetric)	3297	-	-	3350-3180 [7,8]<br>3330-3060 [8]
**3**	alkane	ν_{C-H} stretching (asymmetric) C-H (sp3)	2929	2913<br>2895	2900	3000-2840 [7]<br>2926-2853 [8]
		ν_{C-H} stretching (symmetric) C-H (sp3)	2876	2876	2858	
**4**	amide	$\nu_{N-C=O}$ stretching (C=O) (amide band I)	1657	-	-	1680-1630 [7]<br>1650 [8]
**5**	alcohol	ν_{O-H} stretching O-H (water content)	-	1642	1643	1580-1700 [9]
**6**	amide	δ_{N-H} angular deformation N-H (no plano) (amide band II)	1544	-	-	1570-1515 [8]
	amide	δ_{C-N} angular deformation C-N (amide band II)		-	-	~1550 [7]
**7**	amide	ν_{C-N} stretching C-N	1441	-	-	~1400 [7,8]
**8**	alcohol	δ_{c-O-H} angular deformation C-O-H	1386<br>1321	1415<br>1381<br>1322	1431<br>1375<br>1338<br>1318	1420-1330 [8]<br>1440-1220 [7]
**9**	alcohol (phenol)	ν_{C-O} stretching C-O (phenol)	1245	-	-	~1220 [7]
	ether	ν_{C-O-C} stretching C-O-C (asymmetric)		1244	1281<br>1235	1275-1200 [7]<br>~1250 [7]
**10**	ether	ν_{C-O-C} stretching (asymmetric) C-O-C	1182<br>1143	1182<br>1143	1166	1300-1000 [7]<br>~1120 [7]<br>~1125 [8]
	alkane	δ_{C-H} angular deformation (asymmetric) C-H (sp3) (off plane)				~1150 [8]
	ether (acetal)	ν_{C-O-C} stretching (asymmetric) C-O-C				1200-1020 [7]
**11**	amide	δ_{N-H} angular deformation N-H	1124<br>1087<br>1069	-	-	~1125 [7]
	alcohol (2nd)	ν_{C-O} stretching C-O		1124<br>1087<br>1068	1114<br>1059	1100 [7]<br>1124-1050 [8]
**12**	ether	ν_{C-O-C} stretching C-O-C (symmetric)	1034<br>1011	1034<br>1012	1031	~1040 [7]
	alcohol	ν_{C-O} stretching C-O				1260-1000 [7]
**13**	alcohol (1st)	ν_{C-O} stretching C-O	939	939	-	<1050 [8]
**14**	ether	ν_{C-O-C} stretching (symmetric)	894<br>872<br>807	894<br>872<br>806	895	~850 [7]
**15**	alkane	δ_{C-H} angular deformation (asymmetric) C-H (sp2)	760<br>646<br>606	777<br>761<br>649<br>608	701<br>668<br>613	720 [7,8]
	alcohol	δ_{O-H} angular deformation O-H (off plane)				~650 [7]
	amide	δ_{C-N} angular deformation C-N (symmetric) (off plane)		-	-	800-666 [8]
	amide	δ_{N-H} angular deformation N-H		-	-	750-600 [7]

Table S7. Representative absorptions observed by FTIR of the pretreated samples.

			wavenumber (cm^{-1})						
**region N⁰**	**functional groups**	**assignments**	BM0	BM30	BM60	BM120	BM180	BM240	reference[a]
**1**	alcohol	$\nu_{O\text{-}H}$ stretching O-H (intramolecular)	3385	3379	3395	3399	3408	3426	3400-3300 [7]<br>3550-3200 [8]
**2**	amide	$\nu_{N\text{-}H}$ stretching N-H (asymmetric and symmetric)	3297	3284	3303	3297	3284	3253	3350-3180 [7,8]<br>3330-3060 [8]
**3**	alkane	$\nu_{C\text{-}H}$ stretching (asymmetric) C-H (sp3)	2929	2929	2928	2927	2928	2927	3000-2840 [7]<br>2926-2853 [8]
		$\nu_{C\text{-}H}$ stretching (symmetric) C-H (sp3)	2876	2877	2875	2871	2877	2875	
**4**	amide	$\nu_{N\text{-}C=O}$ stretching (C=O) (amide band I)	1657	1656	1658	1658	1659	1658	1680-1630 [7]<br>1650 [8]
**6**	amide	$\delta_{N\text{-}H}$ angular deformation N-H (no plano) (amide band II)	1544	1544	1544	1545	1547	1545	1570-1515 [8]
	amide	$\delta_{C\text{-}N}$ angular deformation C-N (amide band II)							~1550 [7]
**7**	amide	$\nu_{C\text{-}N}$ stretching C-N	1441	1441	1442	1448	1445	1448	~1400 [7,8]
**8**	alcohol	$\delta_{c\text{-}O\text{-}H}$ angular deformation C-O-H	1386<br>1321	1385<br>1321	1384<br>1319	1404<br>1309	1408<br>1384	1411<br>1384<br>1319	1420-1330 [8]<br>1440-1220 [7]
**9**	alcohol (phenol)	$\nu_{C\text{-}O}$ stretching C-O (phenol)	1245	1246	1247	1250	1249	1249	~1220 [7]
	ether	$\nu_{C\text{-}O\text{-}C}$ stretching (asymmetric) C-O-C							1275-1200 [7]<br>~1250 [7]
**10**	ether	$\nu_{C\text{-}O\text{-}C}$ stretching (asymmetric) C-O-C	1182<br>1143	1144	1146	1147	1147	1146	1300-1000 [7]<br>~1120 [7]<br>~1125 [8]
	alkane	$\delta_{C\text{-}H}$ angular deformation (asymmetric) C-H (sp3) (off plane)							~1150 [8]
	ether (acetal)	$\nu_{C\text{-}O\text{-}C}$ stretching (asymmetric) C-O-C							1200-1020 [7]
**11**	amide	$\delta_{N\text{-}H}$ angular deformation N-H	1124<br>1087<br>1069	1123<br>1087<br>1067	1121<br>1087<br>1059	1119<br>1091<br>1057	1123<br>1091<br>1056	1121<br>1087<br>1062	~1125 [7]
	alcohol (2nd)	$\nu_{C\text{-}O}$ stretching C-O (C2 e C3)							~1100 [7]<br>1124-1050 [8]
**12**	ether	$\nu_{C\text{-}O\text{-}C}$ stretching C-O-C (symmetric)	1034	1034	1032	1029	1028	1031	~1040 [7]
	alcohol	$\nu_{C\text{-}O}$ stretching C-O	1011	1013	1012			1010	1260-1000 [7]
**13**	alcohol (1st)	$\nu_{C\text{-}O}$ stretching C-O	939	940	940	-	-	943	<1050 [8]
**14**	ether	$\nu_{C\text{-}O\text{-}C}$ stretching (symmetric)	894<br>872<br>807	894<br>872<br>808	896<br>872<br>812	896<br>872<br>814	897<br>872<br>814	898<br>872<br>812	~850 [7]
**15**	alkane	$\delta_{C\text{-}H}$ angular deformation (asymmetric) C-H (sp2)	760<br>646<br>606	761<br>648<br>607	764<br>665<br>608	765<br>665<br>606	765<br>661<br>606	763<br>661<br>606	~720 [7,8]
	alcohol	$\delta_{O\text{-}H}$ angular deformation O-H (off plane)							~650 [7]
	amide	$\delta_{C\text{-}N}$ angular deformation C-N symmetric (off plane)							800-666 [8]
	amide	$\delta_{N\text{-}H}$ angular deformation N-H							750-600 [7]

Table S8. Comparison of diffraction peaks observed for mannan I and cellulose I.

2Θ (*more intense peaks)									
**Miller indices**	**101**	**011**	**110***	**111**	**200***	**210**	**211**	**120**	**004/203**
MB0 (this work)	-		16.1	18.4	20.2	23.7	25.2	26.7	33.1
Mannan I (10)	-		16.0	18.2	20.0	23.7	25.2	26.8	-
Mannan I (11)	-		15.9	18.2	19.9	23.5	25.0	26.8	-
Mannan I (12)	12.9		16.19	18.0	20.0	24.0	26.0	27.0	-
Mannan I (13)	13.1	14.9	15.9	18.1	20.0	23.6	25.2	26.7	33.0
Cellulose I (14)	-		16	-	22	-	-	-	35
Cellulose I (15)	-		16.0	18.1	19.5;22	-	-	-	34.0
Cellulose I (16)	-	15	16.5	-	22.8	-	-	-	-
Cellulose I (17)	-	-	15.06	-	22.51	-	-	-	34.63

REFERENCES

(1) Breitmaier, E.; Voelter, W. Carbon-13 NMR Spectroscopy. High-Resolution Methods and Applications in Organic Chemistry and Biochemistry, 3rd ed.; CVH, **1990**.

(2) Nishiyama, M.; Lucas-Torres, C.; Piao, R.; Yanagisawa, Y.; Nishiyama, Y.; Wong, A. Supplemental Shimming for HR-μMAS NMR Spectroscopy. Appl. Magn. Reson. **2019**, 50:1305–1313. https://doi.org/10.1007/s00723-019-01150-8.

(3) Roslund, M.U.; Tahtinen, P.; Niemitz, M.; Sjoholm, R. Complete assignments of the 1H and 13C chemical shifts and JH,H coupling constants in NMR spectra of D-glucopyranose and all D-glucopyranosyl-D-glucopyranosides. Carbohydr. Res. **2008**, 343, 101–112. https://doi.org/10.1016/j.carres.2007.10.008.

(4) Kosaka, A.; Aida, M.; Katsumoto, Y. Reconsidering the activation entropy for anomerization of glucose and mannose in water studied by NMR spectroscopy. J. Mol. Struct. **2015**, 1093, 195–200. http://dx.doi.org/10.1016/j.molstruc.2015.03.038.

(5) Barclay, T.; Ginic-Markovic, M. ; Johnston, M.R. ; Cooper, P. ; Petrovsky, N. Observation of the keto tautomer of D-fructose in D2O using 1H NMR spectroscopy. Carbohydr. Res. **2012**, 347, 136–141. https://doi.org/10.1016/j.carres.2011.11.003.

(6) Bonnet, V.; Duval, R.; Rabiller, C. Oxidation of galactose and derivatives catalysed by galactose oxidase: structure and complete assignments of the NMR spectra of the main product. J. Mol. Catal. B: Enzym. **2003**, 24–25 9–16. DOI:10.1016/S1381-1177(03)00065-1.

(7) Pavia, D.; Lampman, G.M.; Kriz, G.; Vyvyan, J. Introdução a Espectroscopia, 4th ed.; Cengage Learning, **2010**; pp 15-82. ISBN-13: 978-85-221-0708-7. ISBN-10: 85-221-0708-4.

(8) Silverstein, R.M.; Webster, F.X.; Kiemle, D.J. Identificação Espectroscópica de Compostos Orgânicos, 7th ed.; LTC, **2006**; pp 70-104, ISBN 978-85-216-1521-7.

(9) Velazquez, G.; Herrera-Gómez, A.; Polo-Martín, M.O. Identification of bound water through infrared spectroscopy in methylcellulose. J. Food Eng. **2003**, 59, 79–84.

(10) Silva, Y.F.; Alencastro, F.S. ; Souza, N.D. ; Oliveira, R.N. ; Simao, R.A. Investigating the origin of laser-induced fluorescence in mannan-rich Phytelephas macrocarpa seeds before and after thermal aging. Carbohydr. Polym. **2023**, 308, 120632. https://doi.org/10.1016/j.carbpol.2023.120632.

(11) Grimaud, F.; Pizzut-Serin, S.; Tarquis, L.; Ladevèze, S.; Morel, S.; Putaux, J.; Potocki-Veronese, G. In Vitro Synthesis and Crystallization of β1,4-Mannan. Biomacromolecules **2019**, 20, 846−853. https://doi.org/10.1021/acs.biomac.8b01457.

(12) Gibril, M.E.; Zhang, N.; Yi, Y.; Liu, P.; Wang, S.; Tesfaye, T.; Kong, F. Physicochemical characterization and future beneficiation routes of wild fruit waste (Hyphaene Thebaica seed) as a source to extract mannan. J. Clean. Prod. **2020**, 267, 121949. https://doi.org/10.1016/j.jclepro.2020.121949.

(13) Yui, T.; Miyawaki, K.; Yada, M.; Ogawa, K. An evaluation of crystal structure of mannan I by X-ray powder diffraction and molecular mechanics studies. Int. J. Biol. Macromol. **1997**, 21, 243–250. PII S0141-8130(97)00069-X.

(14) Ji, G.; Han, L.; Gao, C.; Xiao, W.; Zhang, Y.; Cao, Y. Quantitative approaches for illustrating correlations among the mechanical fragmentation scales, crystallinity and enzymatic hydrolysis glucose yield of rice straw. Bioresour. Technol. **2017**, 241, 262–268. http://dx.doi.org/10.1016/j.biortech.2017.05.062.

(15) Gupta, V.; Ramakanth, D.; Verma, C.; Maji, P.K.; Gaikwad, K.K. Isolation and characterization of cellulose nanocrystals from amla (Phyllanthus emblica) pomace. Biomass Convers. Biorefin. **2023**, 13, 15451–15462. https://doi.org/10.1007/s13399-021-01852-9.

(16) Terinte, N.; Ibbett, R.; Schuster, K.C. Overview on native cellulose and microcrystalline cellulose I studied by X-ray diffraction (WAXD): Comparison between measurement techniques. Lenzinger Ber. **2011**, 89, 118-131.

(17) Wang, X.; Wu, X.; Guo, K.; Ren, J.; Lin, Q.; Li, H.; Wang, X.; Liu, S. Efficient MicrowaveAssisted Hydrolysis of Microcrystalline Cellulose into Glucose Using New CarbonBased Solid Catalysts. Catal. Lett. **2020**, 150, 138–149. https://doi.org/10.1007/s10562-019-02912-6.
